# Revolutionizing biomedicine: advancements, applications, and prospects of nanocomposite macromolecular carbohydrate-based hydrogel biomaterials: a review

**DOI:** 10.1039/d3ra07391b

**Published:** 2023-12-04

**Authors:** Dalal Mohamed Alshangiti, Tasneam K. El-damhougy, Ahmed Zaher, Mohamed Madani, Mohamed Mohamady ghobashy

**Affiliations:** a College of Science and Humanities, Imam Abdulrahman Bin Faisal University Jubail Saudi Arabia mmadani@iau.edu.sa; b Department of Chemistry, Faculty of Science (Girls), Al-Azhar University P.O. Box: 11754, Yousef Abbas Str. Nasr City Cairo Egypt; c Chemistry Department, Faculty of Science, El-Mansoura University Egypt; d Radiation Research of Polymer Chemistry Department, National Center for Radiation Research and Technology (NCRRT), Atomic Energy Authority P.O. Box 29 Nasr City Cairo Egypt Mohamed.ghobashy@eaea.org.eg Mohamed_ghobashy@yahoo.com

## Abstract

Nanocomposite hydrogel biomaterials represent an exciting Frontier in biomedicine, offering solutions to longstanding challenges. These hydrogels are derived from various biopolymers, including fibrin, silk fibroin, collagen, keratin, gelatin, chitosan, hyaluronic acid, alginate, carrageenan, and cellulose. While these biopolymers possess inherent biocompatibility and renewability, they often suffer from poor mechanical properties and rapid degradation. Researchers have integrated biopolymers such as cellulose, starch, and chitosan into hydrogel matrices to overcome these limitations, resulting in nanocomposite hydrogels. These innovative materials exhibit enhanced mechanical strength, improved biocompatibility, and the ability to finely tune drug release profiles. The marriage of nanotechnology and hydrogel chemistry empowers precise control over these materials' physical and chemical properties, making them ideal for tissue engineering, drug delivery, wound healing, and biosensing applications. Recent advancements in the design, fabrication, and characterization of biopolymer-based nanocomposite hydrogels have showcased their potential to transform biomedicine. Researchers are employing strategic approaches for integrating biopolymer nanoparticles, exploring how nanoparticle properties impact hydrogel performance, and utilizing various characterization techniques to evaluate structure and functionality. Moreover, the diverse biomedical applications of these nanocomposite hydrogels hold promise for improving patient outcomes and addressing unmet clinical needs.

## Abstract 2

Nanocomposite hydrogel biomaterials represent an exciting frontier in biomedicine, offering solutions to longstanding challenges.

## Graphical abstract

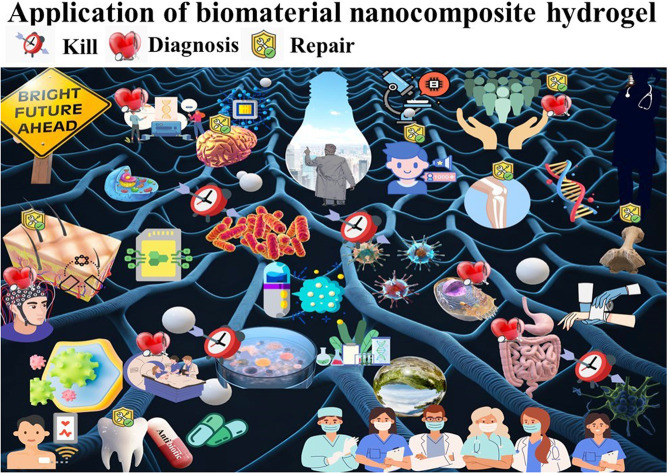

## 1. Introduction

The field of biomedicine has witnessed a revolutionary transformation over recent years, driven by the convergence of cutting-edge technologies and innovative materials. One of the most promising frontiers within this transformative landscape is the emergence of nanocomposite hydrogel biomaterials, which hold remarkable potential for a diverse array of biomedical applications.^1^ By seamlessly integrating nanoparticles within hydrogel matrices, these nanocomposite hydrogels exhibit a harmonious fusion of properties and functionalities that has the potential to reshape the boundaries of modern healthcare.^2^

Nanocomposite hydrogel biomaterials represent a significant leap forward in biomaterial design and engineering, offering a versatile platform with many advantages.^3^ The ingenious incorporation of nanoparticles into hydrogel networks imparts these biomaterials with unique characteristics, ranging from enhanced mechanical strength and improved biocompatibility to precisely tailored drug release profiles.^4^ This amalgamation of nanotechnology and hydrogel chemistry presents an unprecedented opportunity to fine-tune biomaterials' physical, chemical, and biological attributes, thereby enabling tailored solutions for complex biomedical challenges.

Recent work has spotlighted the continuous evolution of advanced hydrogel systems for diverse biomedical applications. An injectable antibacterial hydrogel composed of quaternized chitosan and alginate developed by Zhang *et al.*^5^ demonstrates that a small molecule phosphorus-based compound like magnesium ascorbyl phosphate (MAP), when delivered using a hydrogel scaffold, has the potential for effective bone defect repair through its multifunctional abilities in antioxidant activity, calcium uptake promotion, and angiogenesis stimulation. Xin *et al.*^6^ showed that natural okra-based hydrogels have potential as skin regeneration materials and tissue engineering applications due to their ability to speed up the healing of diabetic wounds. The antioxidant and anti-inflammatory properties of okra help reduce oxidative stress and inflammation, major issues in chronic diabetic wounds.

Zhong *et al.*^7^ developed a novel wound dressing made of electrospun fibers and a hydrogel. By encapsulating the drug deferoxamine, the dressing was able to promote angiogenesis, antioxidant activity, and wound healing in diabetic rats. The dressing has the potential for treating chronic wounds in diabetic patients. Hu *et al.*^8^ developed a novel double-sensitive composite hydrogel, incorporating tetrasulfide-bridged mesoporous silica (4S-MSNs), which addresses critical challenges in the postsurgical treatment of tumors. This hydrogel, integrated into an oxidized dextran/chitosan network, enhances mechanical strength and offers dual pH/redox sensitivity for efficient and safe drug delivery. The 4S-MSNs hydrogel maintains the advantageous physicochemical properties of polysaccharide hydrogels, including high hydrophilicity, potent antibacterial activity, and excellent biocompatibility. This innovative approach holds promise in mitigating tumor recurrence and preventing wound microbial infections, presenting a multifunctional strategy for enhanced postsurgical therapy. In tissue engineering, nanocomposite hydrogel biomaterials have ignited a paradigm shift. These materials can potentially revolutionize how we approach tissue regeneration and repair.^9^ Creating scaffolds with controlled mechanical properties, biodegradability, and bioactive cues is invaluable for guiding cellular behavior and tissue growth.^10^ Stem cell differentiation, for instance, can be directed within nanocomposite hydrogel matrices, unlocking new avenues for regenerating functional tissues and organs.^11^ Nanocomposite hydrogels' intricately designed hybrid nature brings us closer to achieving the long-standing dream of creating artificial organs and tissues seamlessly integrating with the human body.^12^

The capabilities of nanocomposite hydrogel biomaterials equally impact the realm of drug delivery. These biomaterials offer a dynamic platform for designing novel drug delivery systems that provide spatial and temporal control over therapeutic release.^13^ By harnessing nanoparticles' unique properties and hydrogels' tunable nature, researchers can engineer systems that respond to specific cues within the body, releasing therapeutic agents on demand.^14^ The potential applications span a broad spectrum, from cancer therapies where precise drug targeting can minimize side effects to chronic disease management through sustained and controlled drug release.^15^

Wound healing, a fundamental aspect of healthcare, is also revolutionized by nanocomposite hydrogel biomaterials.^16^ Incorporating nanoparticles imparts these hydrogels with antimicrobial properties, accelerating the healing process and reducing the risk of infections.^17^ Moreover, the mechanical strength of nanocomposite hydrogels enhances their suitability for wound dressings, supporting the injured area while promoting tissue regeneration. The synergistic combination of enhanced mechanical properties and tailored drug release kinetics ensures that these biomaterials address multiple facets of wound care, resulting in improved therapeutic outcomes.^18^

This comprehensive review embarks on a journey to explore the multifaceted realm of nanocomposite hydrogel biomaterials, delving into their design, fabrication, characterization, and applications. Through an in-depth examination of these biomaterials, we aim to provide a holistic understanding of their transformative potential in biomedicine. By scrutinizing the intricate interplay between nanoparticles and hydrogels and delving into their diverse biomedical applications, we hope to shed light on leveraging these materials to improve patient outcomes, advance healthcare, and address global challenges in regenerative medicine and beyond.

The rapid evolution of biomaterials science has catalyzed transformative breakthroughs in biomedicine. Among these innovations, nanocomposite macromolecular carbohydrate-based hydrogels have emerged as a promising class of materials, offering exceptional versatility, biocompatibility, and tunable properties. This comprehensive review explores the recent advancements, multifaceted applications, and exciting prospects of these hydrogel biomaterials in revolutionizing various facets of biomedicine.

### 1.1. Advancements

We delve into the synthesis and engineering strategies that have enabled the development of nanocomposite macromolecular carbohydrate-based hydrogels. This includes the incorporation of nanomaterials (*e.g.*, nanoparticles, nanofibers) and bioactive molecules (*e.g.*, growth factors, enzymes) to enhance mechanical strength, bioactivity, and controlled drug release. Moreover, the design of responsive hydrogels capable of adapting to environmental cues, such as pH, temperature, and enzymatic activity, is discussed.

### 1.2. Applications

Highlighting the versatility of these hydrogel biomaterials, we explore their applications in tissue engineering, drug delivery, wound healing, regenerative medicine, and diagnostics. Specific examples include using carbohydrate-based hydrogels as scaffolds for tissue regeneration, carriers for targeted drug delivery, wound dressings with antimicrobial properties, and platforms for bioimaging and biosensing.

### 1.3. Prospects

The review outlines the future prospects and potential directions for nanocomposite macromolecular carbohydrate-based hydrogels in biomedicine. This encompasses integrating cutting-edge technologies like 3D bioprinting and microfluidics for precision medicine and personalized therapy. Furthermore, we discuss the prospects of harnessing these hydrogels for combating emerging healthcare challenges, such as infectious diseases and neurodegenerative disorders.

This review illuminates the profound impact of nanocomposite macromolecular carbohydrate-based hydrogels on the biomedical landscape through a critical synthesis of the current literature and research trends. These biomaterials offer a unique synergy of biocompatibility, tunability, and multifunctionality, making them pivotal players in the quest for innovative solutions to complex biomedical challenges. As we navigate the uncharted territory of biomedicine, these hydrogel biomaterials stand as trailblazers, poised to redefine the boundaries of therapeutic interventions and diagnostic technologies.

## 2. Nanocomposite hydrogel formulation and design

In the ever-evolving landscape of biomaterials, the emergence of nanocomposite hydrogels is a testament to human ingenuity and the power of interdisciplinary innovation. These remarkable materials, born from the marriage of nanotechnology and hydrogel chemistry, are poised to revolutionize the field of biomedicine. Nanocomposite hydrogels, with their unique blend of properties and functionalities, promise to address critical challenges in diverse biomedical applications, ranging from tissue engineering and drug delivery to wound healing and biosensing.

At the heart of nanocomposite hydrogel innovation lies a meticulous process of formulation and design that capitalizes on the synergistic interplay between nanoparticles and hydrogel matrices.^19^ This intricate dance between nanoscale entities and hydrophilic networks is critical to unlocking enhanced mechanical strength, tailored drug release profiles, and improved biocompatibility attributes vital for addressing complex biomedical needs.^20^ The formulation of nanocomposite hydrogels represents a symphony of scientific disciplines, each contributing a unique note to the harmonious composition of these materials.^21^ At its core, the design process aims to orchestrate the perfect blend of nanoparticle characteristics and hydrogel properties, yielding a biomaterial that transcends the capabilities of its components. The formulation and design of nanocomposite hydrogels depend on several factors, such as the type and concentration of nanomaterials, the polymer composition and crosslinking method, the solvent and pH conditions, and the desired application. Some of the common nanomaterials used in nanocomposite hydrogels are carbon nanotubes, graphene, metal nanoparticles, metal oxides, quantum dots, and clay. These nanomaterials can be dispersed uniformly or selectively within the hydrogel matrix or attached to the polymer chains or crosslinkers. The nanomaterials can also interact with the polymer chains through physical or chemical bonds, affecting the hydrogel's swelling behavior and mechanical properties. The formulation and design of nanocomposite hydrogels require careful optimization to achieve the desired performance and functionality. Pereira *et al.*^18^ investigate the synthesis and characterization of hydrogel nanocomposites based on partially hydrolyzed polyacrylamide, polyethyleneimine, and modified clay. The nanocomposites were developed to be used as sealing agents in high water-producing zones in oil fields. The conventional hydrogels and nanocomposite hydrogels with unmodified and organic modified bentonite clay were fabricated. The nanocomposite hydrogels showed higher elastic modulus and stability than the conventional hydrogels, indicating the reinforcing effect of clay. The organic-modified clay nanocomposite performed better due to its stronger interactions with the polymer matrix. The cross-linker concentration also influenced the gelation kinetics and properties of the hydrogels.

Bovone *et al.*^22^ discuss a strategy to reinforce and expand polymer-nanoparticle (PNP) hydrogels. PNP hydrogels are formed by mixing polymer chains and nanoparticles and can exhibit shear-thinning and self-healing properties. However, the range of mechanical properties of PNP hydrogels is limited due to the specific interactions between select polymers and nanoparticles. The researchers used alpha-cyclodextrin (α-CD) as a supramolecular motif to enhance the mechanical properties of PNP hydrogels. The wrapping of α-CD onto the polyethylene glycol chains on the nanoparticle surface resulted in polypseudorotoxane formation, increasing nanoparticle jamming and interactions. The addition of α-CD increased the storage modulus of the hydrogels by orders of magnitude while maintaining shear-thinning and self-healing properties. Importantly, the use of α-CD enabled modular design by allowing the exchange of the polymer and nanoparticle components. The researchers formulated hydrogels with different polymers for 3D printing, drug delivery, and functional nanoparticles to engineer conductive or magnetic materials. Nanocomposite hydrogels have been categorized into four groups based on the type of nanoparticles they incorporate carbon-based, polymeric-based, inorganic-based, and metallic-based hydrogels. These categorizations in Table 1 stem from nanoparticles' diverse nature and potential interactions within the hydrogel matrix, leading to a wide range of material properties and applications.

**Table 1:** Highlights the characteristics and applications of nanocomposite hydrogels based on the type of their nanoparticles: carbon-based, polymeric-based, inorganic-based, and metallic-based hydrogels

Category	Nanoparticle types	Characteristics	Applications
Carbon-based nanocomposite hydrogels	Carbon nanotubes (CNTs), graphene, graphene oxide	Exceptional mechanical, electrical, and thermal properties	Tissue engineering scaffolds
		Improved strength, conductivity, and thermal stability	Flexible electronics sensors
Polymeric-based nanocomposite hydrogels	Nanofibers, micelles, dendrimers	Structural support, enhanced drug encapsulation, regulated release kinetics	Drug delivery
			Wound healing
			Tissue regeneration
Inorganic-based nanocomposite hydrogels	Silica nanoparticles, hydroxyapatite, metal oxides	Modulated mechanical strength, improved bioactivity, controlled ion release	Bone tissue engineering
			Dental materials
			Controlled drug delivery
			Antimicrobial agent
Metallic-based nanocomposite hydrogels	Gold nanoparticles, silver nanoparticles	Distinct optical, antimicrobial, photothermal, and catalytic properties	Wound dressings
		Enhanced antimicrobial activity, localized drug release	Cancer therapy
			Antibacterial applications

### 2.1. Carbon-based nanocomposite hydrogels

Carbon-based nanoparticles, such as carbon nanotubes (CNTs) and graphene derivatives, have gained significant attention for their exceptional mechanical, electrical, and thermal properties. Incorporating these nanoparticles into hydrogels enhances strength, electrical conductivity, and thermal stability. Carbon-based nanocomposite hydrogels find applications in tissue engineering scaffolds, flexible electronics, and sensors. Nanocomposite hydrogels (NCHs) from carbon-based nanomaterials such as carbon nanotubes (CNTs) and graphene. CNTs exist in different atomic configurations (namely armchair and zig-zag) and architectures (single- and multi-walled) and can be chemically modified to enhance their hydrophilicity and, therefore, their interaction with the surrounding hydrogel.^23^ Based on the search results, there are some common methods for synthesizing carbon-based nanocomposite hydrogels. *In situ polymerization*: this method involves the polymerization of monomers in the presence of carbon nanoparticles to form a hydrogel. The carbon nanoparticles can be incorporated into the hydrogel matrix during polymerization.^24^*Crosslinking*: carbon nanoparticles can be crosslinked with a polymer matrix to form a hydrogel. This method involves mixing the carbon nanoparticles with a polymer solution and then crosslinking the mixture to form a hydrogel.^25^*Freeze-drying*: freeze-drying is a technique that can synthesize carbon-based nanocomposite hydrogels.^26^ This method involves freezing a solution containing carbon nanoparticles and a polymer, then drying the mixture under a vacuum to form a hydrogel. *Electrospinning*: electrospinning can also synthesize carbon-based nanocomposite hydrogels.^27^ This method involves using an electric field to spin a polymer solution containing carbon nanoparticles into nanofibers, which can then be crosslinked to form a hydrogel. The addition/conjugation of CNTs and graphene derivatives provides NCHs with improved mechanical properties and electrical conductivity. For these reasons, NCHs embedding carbon-based nanomaterials can potentially be used for numerous applications, such as tissue engineering of electrically conductive tissues and electrically stimulated drug delivery. Biocompatible nanomaterials have attracted enormous interest in biomedical applications. Carbonaceous materials, including carbon nanotubes (CNTs), have been widely explored in wound healing and other applications because of their superior physicochemical and potential biomedical properties at the nanoscale level. CNTs-based hydrogels are widely used for wound-healing and antibacterial applications.^28^ To synthesize CNT-hydrogel nanocomposites, CNTs are usually integrated into hydrogel precursor solutions and polymerized/crosslinked to form a hydrogel network structure. The CNTs are dispersed and embedded within the hydrogel matrix. Carbon-based nanocomposite hydrogels have a wide range of potential applications beyond energy-related fields. Belmonte *et al.*^29^ investigated the impact of graphene oxide lateral dimensions on the properties of methacrylate gelatin hydrogels. Graphene oxide sheets were derived from the oxidation of commercial graphene and used to fabricate the hydrogel composites *via* photopolymerization with methacrylate gelatin. The researchers found that increasing the graphene oxide sheet sizes improved the mechanical strength of the hydrogels, with higher compressive modulus and low mechanical hysteresis under 10%. This indicated low mechanical energy dissipation even after multiple deformation cycles. The nanocomposite hydrogels also demonstrated antibacterial effects against two clinical drug-resistant bacterial strains, primarily through contact. Eco-friendly materials,^30,31^ bioplastics derived from renewable sources reduce reliance on fossil fuels, bio-based materials, including semi-permeable membrane,^32^ blend polymer.^33^ Eco-friendly materials aims to use as green renewable resources of to protect the environment from negative effects.^34–36^ Bio-based polymers and natural, are derived from renewable resources and often require less energy.^37^ Larger graphene oxide lateral dimensions increased the antibacterial capacity against Gram-negative bacteria. The study demonstrated that the graphene oxide sheets' lateral dimensions significantly impacted the hydrogels' properties, including mechanical, rheological, thermal, and antibacterial properties. Here are some potential applications of carbon-based nanocomposite hydrogels: *biomedical applications*: carbon-based nanocomposite hydrogels show promise in various biomedical applications. They can be used for drug delivery systems, tissue engineering scaffolds, wound healing dressings, and biosensors. Carbon nanomaterials in hydrogels offer advanced biomaterials for medical applications.^38^*Biomedical imaging*: carbon-based nanocomposite hydrogels can be utilized for biomedical imaging applications. Incorporating carbon nanoparticles into hydrogels can enhance their imaging capabilities, enabling better visualization and diagnosis of diseases. *Controlled release systems*: carbon-based nanocomposite hydrogels can be designed as smart materials for controlled drug release. The unique properties of carbon nanoparticles, such as high surface area and tunable surface chemistry, allow for precise control over drug release kinetics. *Tissue engineering*: carbon-based nanocomposite hydrogels have potential applications in tissue engineering.^39^ They can provide a suitable cell growth and differentiation environment, promoting tissue regeneration. Carbon-based nanocomposite hydrogels' mechanical properties and biocompatibility make them attractive for scaffold materials in tissue engineering. *Biosensors*: carbon-based nanocomposite hydrogels can be utilized to develop biosensors.^40^ Incorporating carbon nanoparticles into hydrogels can enhance the sensitivity and selectivity of biosensors, enabling the detection of specific biomarkers or analytes. *Biocompatible coatings*: carbon-based nanocomposite hydrogels can be used as biocompatible coatings for medical devices. These coatings can improve the biocompatibility and functionality of medical implants, reducing the risk of adverse reactions and promoting better integration with the surrounding tissues. Huang *et al.*^41^ investigated a strategy to enhance the mechanical properties of chitosan-based composite hydrogels. Chitosan is a favorable material for making hydrogels due to its hydrophilic and biocompatible nature. However, achieving robust chitosan-based hydrogels remains challenging. The researchers propose a multiple mechanisms-based network design using graphene oxide-filled chitosan as the first network and polyacrylamide as the second network. This forms a double network composite hydrogel structure. The graphene oxide-filled chitosan network has a multi-structure that enhances its energy dissipation capacity. When combined with the polyacrylamide second network, the coupling effect between the two networks allows for synergistic enhancement of the mechanical properties. The researchers explore the role of the individual networks and find that both are essential. The first network mainly contributes to energy dissipation while the second network plays a role in the synergistic enhancement in the double network composite gels.

### 2.2. Polymeric-based nanocomposite hydrogels

Polymeric nanoparticles, including nanofibers, micelles, and dendrimers, offer a diverse toolkit for tailoring hydrogel properties. These nanoparticles can provide structural support, enhance drug encapsulation, and regulate release kinetics. Polymeric-based nanocomposite hydrogels are utilized in drug delivery, wound healing, and tissue regeneration.^42^ Polymer-based hydrogels are hydrophilic polymer networks with crosslinks widely applied for drug delivery applications because they hold large amounts of water and biological fluids and control drug release based on their unique physicochemical properties and biocompatibility. Current trends in developing hydrogel drug delivery systems involve the release of drugs in response to specific triggers such as pH, temperature, or enzymes for targeted drugs.^14^ The most common methods for synthesizing polymeric-based nanocomposite hydrogels include *in situ polymerization*. This method involves the polymerization of monomers and the incorporation of nanoparticles within the hydrogel matrix.^43^ The nanoparticles can be added during the polymerization process, allowing for the formation of a nanocomposite hydrogel. *Crosslinking*: nanocomposite hydrogels can be synthesized by crosslinking pre-formed polymers with nanoparticles.^44^ The nanoparticles are mixed with the polymer solution, and crosslinking agents form a three-dimensional network. *Blending approach*: in this method, pre-formed nanoparticles and polymers are blended to form a nanocomposite hydrogel.^44^ The nanoparticles and polymers can be mixed using stirring, sonication, or extrusion techniques. The radical polymerization method involves using radical initiators to polymerize monomers in the presence of nanoparticles.^45^ The nanoparticles are dispersed within the monomer solution, and the polymerization is initiated to form the hydrogel network. High-energy irradiation method: nanocomposite hydrogels can be synthesized by subjecting a mixture of polymers and nanoparticles to high-energy irradiation, such as gamma rays or electron beams.^46^ The irradiation induces crosslinking between the polymers and the nanoparticles, resulting in the formation of a hydrogel. *Enzyme-driven synthesis method*: this method utilizes enzymes to catalyze the polymerization of monomers in the presence of nanoparticles.^47^ The enzymes act as catalysts to facilitate the formation of the hydrogel network. These methods offer different approaches to incorporating nanoparticles into polymeric hydrogels, allowing for synthesizing nanocomposite hydrogels with tailored properties. The choice of method depends on factors such as the desired properties of the hydrogel, the type of nanoparticles, and the specific application requirements. Yang *et al.*^48^ created an injectable nanocomposite hydrogel to promote wound healing for infected chronic diabetic wounds. The hydrogel has several functions that help with this. First, it's made up of components that can kill bacteria. It eliminated over 99.99% of *E. coli* and *Staphylococcus aureus* bacteria in tests. Second, the hydrogel has photo-thermal properties. This means it can generate heat when exposed to light, which can help kill bacteria and promote wound healing. Third, the hydrogel has antioxidant properties. It showed a free radical scavenging capability of over 70% in tests. The researchers tested the hydrogel in wound healing experiments on animals. They found that the hydrogel promoted healing better than a commercially available wound dressing. It helped prevent infection, reduced inflammation, supported collagen deposition, and improved tissue formation at the wound sites. In summary, the multifunctional hyaluronic acid-based injectable nanocomposite hydrogel showed potential as a wound dressing for infected chronic diabetic wounds due to its ability to kill bacteria, generate heat, reduce free radicals, and promote wound healing in tests.

### 2.3. Inorganic-based nanocomposite hydrogels

Inorganic nanoparticles, such as silica, hydroxyapatite, and metal oxides, confer unique physicochemical properties to hydrogels. These nanoparticles can modulate mechanical strength, improve bioactivity, and facilitate controlled ion release. Inorganic-based nanocomposite hydrogels are applied in bone tissue engineering, dental materials, and controlled drug delivery. This approach can be exploited in synthesizing materials that exhibit defined nanoporosity. Inorganic-based nanocomposite hydrogels are promising materials with superior physicochemical and biological properties. They have found broad applicability in various fields of science and technology, including biomedical sciences and engineering. Nanomaterial-filled, hydrated polymeric networks exhibit higher elasticity and strength than traditionally made hydrogels. A range of natural and synthetic polymers are used to design nanocomposite networks. By controlling the interactions between nanoparticles and polymer chains, a range of physical, chemical, and biological properties can be engineered. Combining organic (polymer) and inorganic (clay) structures gives these hydrogels improved physical, chemical, electrical, biological, and swelling/de-swelling properties that cannot be achieved by either material alone. Most inorganic nanoparticles used for nanocomposite hydrogels are already present in and necessary for the body, thus presenting no negative impacts on the body. Some of them, like calcium and silicon, help prevent bone loss and skeletal development. Others, like nano clays, improve hydrogels' structural formation and characteristics. Inorganic nanocomposite hydrogels have been used for bone tissue engineering applications. Incorporating nanomaterials into cell-laden hydrogels is a straightforward tactic for producing tissue engineering structures that integrate perfectly with the body and for tailoring the material characteristics of hydrogels without hindering nutrient exchange with the surroundings. Various nanofillers have been used for designing inorganic nanocomposite hydrogels, including graphene, metallic nanoparticles, clay minerals, and fumed silica. These nanocomposite hydrogels exhibited superior properties and have been used for various biomedical applications, including drug delivery, imaging and gene silencing, and orthopedic applications. Overall, inorganic-based and organic-based nanocomposite hydrogels have unique properties and applications. Inorganic-based hydrogels offer improved physical properties and are particularly advantageous for bone tissue engineering, while organic-based hydrogels have a wide range of applications in drug delivery, artificial muscles, and sensors. The choice between the two depends on the specific requirements of the intended application. Inorganic nanocomposite hydrogels can simulate the cell matrix microenvironment of human bone tissue. They have been used for bone tissue engineering applications, taking advantage of their improved mechanical properties and biocompatibility. Incorporating nanomaterials into cell-laden hydrogels is a straightforward tactic for producing tissue engineering structures that integrate perfectly with the body and for tailoring the material characteristics of hydrogels without hindering nutrient exchange with the surroundings. Inorganic nanocomposite hydrogels have been used to repair bone tissues. They have been shown to modulate biomarkers of bone differentiation and enhance bone mineralization. The most widely used inorganic phosphate-based ceramics for bone tissue engineering are calcium phosphates, including hydroxyapatite (HA), β-tricalcium phosphate (β-TCP), and biphasic calcium phosphate (BCP). Clay-based nanocomposite hydrogels have also shown significant potential for bone tissue engineering. Calcium phosphates: Calcium phosphates, such as hydroxyapatite (HA), β-tricalcium phosphate (β-TCP), and biphasic calcium phosphate (BCP), are widely used inorganic nanoparticles for bone tissue engineering.^49^ They provide a bioactive environment for bone regeneration and enhance the mechanical properties of the hydrogels. Clay-based nanocomposite hydrogels have shown significant potential for bone tissue engineering. Nanoclays, such as montmorillonite and halloysite, have been incorporated into hydrogels to improve their structural formation and characteristics. Silica nanoparticles have reinforced hydrogels for bone tissue engineering applications.^50^ They enhance the mechanical properties and bioactivity of the hydrogels. They can provide antimicrobial properties and enhance the osteogenic differentiation of cells. These inorganic nanoparticles are chosen based on their biocompatibility, bioactivity, and ability to enhance the mechanical properties of the hydrogels. They play a crucial role in improving the performance and functionality of the hydrogels for bone tissue engineering applications. Incorporating inorganic nanoparticles into hydrogels for bone tissue engineering is a promising approach, but it also presents some challenges. Achieving uniform dispersion of inorganic nanoparticles within the hydrogel matrix is challenging.^51^ The nanoparticles tend to agglomerate, leading to inhomogeneous distribution and reduced mechanical properties. The choice of inorganic nanoparticles is critical for the success of the hydrogel. The nanoparticles must be biocompatible, bioactive, and able to enhance the mechanical properties of the hydrogel. The synthesis of inorganic nanocomposite hydrogels requires specific materials and methods. The hydrogels must be designed to have specific properties based on the choice of inorganic nanoparticles and crosslinking methods. The degradation rate of inorganic nanocomposite hydrogels must be controlled to match the rate of tissue regeneration. Rapid degradation can lead to the loss of mechanical properties and insufficient support for tissue regeneration. The inorganic nanoparticles can affect the biological properties of the hydrogel, such as cell adhesion and proliferation. The nanoparticles must be chosen carefully to ensure they do not negatively impact the hydrogel's biological properties. Ghobashy *et al.*^37^ synthesized carbonated hydroxyapatite nanocomposites using sodium hyaluronate-polyacrylamide hydrogel as a template. The hydrogel was prepared using gamma irradiation and acted as a template and carbon source for the hydroxyapatite. The impregnation of the hydrogel in calcium and phosphate solutions at different cycles allowed for controlling the Ca/P ratio and producing different hydroxyapatite compounds after calcination. The sodium hyaluronate was unstable under radiation but was stabilized by mixing with polyacrylamide. This produced a cross-linked hydrogel that provided carbon ions for the carbonated hydroxyapatite. The hydroxyapatite nanocomposites also had minor substitutions of sodium and magnesium ions. The researchers analyzed the effectiveness of the carbonated hydroxyapatite compared to hydroxyapatite for osteoblast cell regeneration. They found that the carbonated hydroxyapatite with a Ca/P ratio of 1.89 showed significantly increased cell differentiation and regeneration compared to the hydroxyapatite control. This indicates that the carbonated hydroxyapatite has improved osteogenic ability. In summary, the authors synthesized carbonated hydroxyapatite nanocomposites using gamma radiation cross-linked hydrogel as a template. The carbonated hydroxyapatite showed better osteoblast cell regeneration than hydroxyapatite, indicating its potential as a biomaterial. The gamma radiation technique allowed for producing a stable and sterilized hydrogel template. Yang *et al.*^52^ discuss a novel method for creating nanocomposite hydrogels-based TiO_2_@MS-SH nanoparticles for sonodynamic therapy. The process involves the utilization of specialized nanoparticles, specifically titanium dioxide nanoparticles coated with mesoporous silica and functionalized with thiol groups. These engineered nanoparticles are referred to as TiO_2_@MS-SH. The researchers employ the TiO_2_@MS-SH nanoparticles as crosslinking agents, interacting with dextran molecules functionalized with norbornene groups. This interaction occurs when exposed to ultrasound irradiation, resulting in the formation of the nanocomposite hydrogel. The TiO_2_@MS-SH nanoparticles have a dual role within the hydrogel structure. Firstly, they function as crosslinkers, enhancing the mechanical properties of the hydrogel when subjected to ultrasound. Secondly, when exposed to ultrasound, these nanoparticles generate reactive oxygen species (ROS), a chemically active molecule. This ROS generation capability makes the hydrogel suitable for use in sonodynamic therapy. The researchers demonstrate that they can fine-tune the characteristics and effectiveness of the hydrogel by adjusting the frequency of the ultrasound used in the crosslinking process. They have created a versatile nanocomposite hydrogel platform through ultrasonic interfacial crosslinking. This platform exhibits significant potential for applications in sonodynamic therapy, a therapeutic approach that utilizes ultrasound and ROS to target and destroy cancer cells or other pathological tissues. The study's findings highlight the potential of this technology for advancing medical treatments and therapy options.

### 2.4. Metallic-based nanocomposite hydrogels

Metallic nanoparticles, such as gold nanoparticles and silver nanoparticles, bring forth distinct optical, antimicrobial, and catalytic properties to hydrogel matrices.^53^ C metallic nanoparticles can enhance antimicrobial activity, photothermal therapy, and localized drug release. Metallic-based nanocomposite hydrogels find utility in wound dressings, cancer therapy, and antibacterial applications. Synthesizing metallic-based nanocomposite hydrogels involves incorporating metallic nanoparticles into the hydrogel matrix. *In situ* synthesis: one approach synthesizes metallic nanoparticles within the hydrogel matrix. This involves incorporating the precursors for metallic nanoparticles during the hydrogel synthesis process, allowing for the simultaneous formation of the hydrogel and nanoparticles. Incorporation of pre-synthesized nanoparticles: metallic nanoparticles, such as gold (Au) and silver (Ag), can be incorporated into hydrogels. These nanoparticles are typically synthesized separately and mixed with the hydrogel matrix during fabrication. Structural modification: factors such as structural modification, material stability, processability, and solubility must be considered during the fabrication or modification of metallic nanocomposite hydrogels. These conditions can vary and impact the cross-linking materials used in the hydrogel. Properties and applications: metallic-based nanocomposite hydrogels offer superior physical, chemical, and biomedical properties compared to conventional polymer hydrogels. They can exhibit electrical/magnetic responsiveness when metallic nanoparticles are included within the hydrogel matrix. These hydrogels have potential applications in various biomedical and pharmaceutical fields. There are several methods for synthesizing metallic-based nanocomposite hydrogels. *In situ* synthesis involves synthesizing the metallic nanoparticles within the hydrogel matrix. The precursors for metallic nanoparticles are incorporated into the hydrogel, allowing for the simultaneous formation of the hydrogel and nanoparticles. Incorporation of pre-synthesized nanoparticles: metallic nanoparticles, such as gold (Au), silver (Ag), and metal oxides (*e.g.*, iron oxide, titania, alumina, zirconia), can be synthesized separately and then mixed with the hydrogel matrix during the fabrication process. The synthesis of metallic nanoparticles can be classified into two primary methodologies: bottom-up and top-down. In the bottom-up method, nanoparticles are synthesized from smaller building blocks, while in the top-down method, larger structures are broken down into smaller nanoparticles. Electrospinning is a technique that can be used to synthesize metallic-based nanocomposite hydrogels. This method involves using an electric field to spin a polymer solution containing metallic nanoparticles into nanofibers, which can then be crosslinked to form a hydrogel. Llorens *et al.*^54^ discuss using metallic-based micro and nanostructured materials in food contact materials and active food packaging. These materials can enhance mechanical strength and barrier properties and provide antimicrobial effects. Silver-based nanoengineered materials are currently the most commonly used due to their antimicrobial capacity. These materials can increase the shelf life of packaged foods by preventing degradation and contamination. Specific metal ions like silver, copper, and zinc have natural antimicrobial properties in nanoparticle form. They can effectively kill bacteria and fungi. They can provide properties like ethylene oxidation and oxygen scavenging to preserve food freshness. The main challenges are safety concerns over the migration of metal ions from the packaging into the food. Proper characterization and testing of these nanomaterials are needed to ensure they are safe for food contact. In summary, metallic nanoparticles show great potential as antimicrobial agents in food packaging to preserve food quality and extend shelf life. However more research is needed to ensure they are used safely and within regulatory guidelines. With further development, these materials could transform the food packaging industry. The categorization of nanocomposite hydrogels based on nanoparticle type underscores these materials' versatility and potential to address a wide array of biomedical challenges. By judiciously selecting and integrating nanoparticles, researchers can tailor the properties of nanocomposite hydrogels to suit specific applications, ranging from tissue engineering to drug delivery and beyond. This classification framework offers a valuable roadmap for researchers exploring the multifaceted realm of nanocomposite hydrogel design.

Nanocomposite macromolecular carbohydrates hydrogel biomaterials hold immense potential in revolutionizing biomedicine. While the provided search results do not directly address this specific topic, I can provide a general overview based on existing knowledge. Biomaterial advancements: biomaterials have seen significant advancements by integrating nanotechnology into their development. This includes using nanocomposite materials that can incorporate macromolecular carbohydrates into hydrogel structures, offering unique properties and applications in the biomedical field. Nanoparticle-based drug delivery systems, which may incorporate hydrogel biomaterials, have shown promise in treating various medical conditions, including cancer. This indicates the potential for nanocomposite macromolecular carbohydrate-based hydrogels in drug delivery. Developing hydrogels, including those with nanocomposite structures, is crucial for various biomedical applications. These hydrogels can exhibit properties such as biocompatibility and flexibility, making them suitable for tissue engineering and regenerative medicine.^6^ While specific details about nanocomposite macromolecular carbohydrate-based hydrogel biomaterials are not available in the provided search results, the combination of nanotechnology, hydrogel science, and biomaterials is a rapidly evolving field with the potential to revolutionize biomedicine in areas like tissue engineering, drug delivery, and more.

**Fig. 1:**
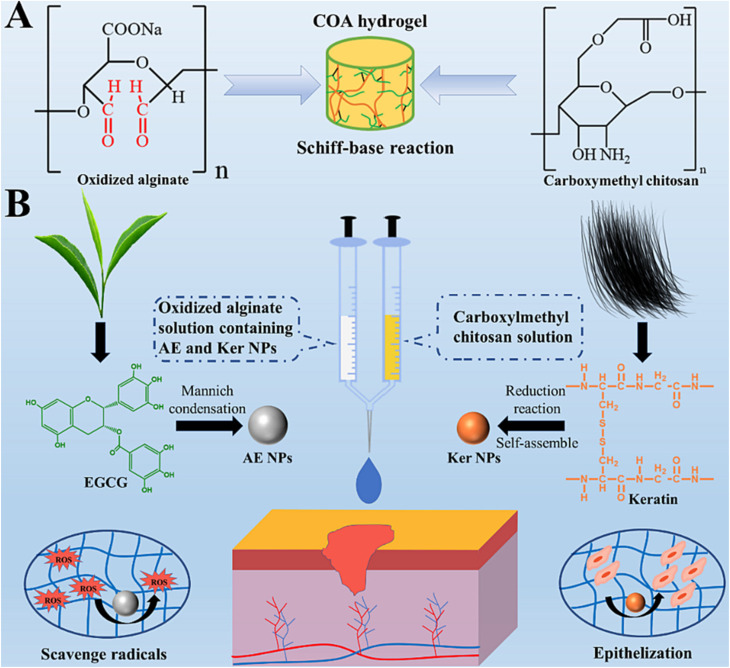
Figure illustrates the fabrication process of injectable hydrogels for wound repair using oxidized alginate and carboxymethyl chitosan. (A) The formation of the fundamental injectable hydrogels through the Schiff-base reaction is depicted. (B) The functionalization of injectable composite hydrogel with Ag-covered EGCG nanoparticles (AE NPs) and keratin nanoparticles (Ker NPs) is presented, showcasing the application for wound repair to scavenge radicals and promote epithelization. Copyright with permission from Elsevier 2022 (ref. 55).

Further research in this area is essential to unlock its full potential. Fig. 1 demonstrates that incorporating nanoparticles into an injectable hydrogel can improve its therapeutic properties for wound healing by endowing it with anti-oxidant and tissue regeneration capabilities.^55^ The functionalized hydrogel showed potential as an advanced wound dressing. An injectable oxidized alginate/carboxymethyl chitosan hydrogel functionalized with nanoparticles for wound repair. The nanoparticles help improve the therapeutic effect of the hydrogel. The synthesized keratin nanoparticles have epithelization capability, and EGCG nanoparticles are covered with silver nanoparticles with radical scavenging capability. These nanoparticles functionalize the oxidized alginate/carboxymethyl chitosan hydrogel.

The radical scavenging experiments showed that the EGCG nanoparticles have anti-oxidative capacity. The hydrogel had a gelation time of around 216 seconds and a storage modulus of 403 Pa. Animal experiments showed that the hydrogel accelerated wound healing, especially in the early stages.

Table 2 gives examples of common polysaccharides-based nanocomposite hydrogel and its applications: Starch is a polysaccharide found in plants and is composed of glucose units. It serves as a storage form of plant energy and is a major component of many staple foods like potatoes, rice, and wheat. Glycogen is the storage form of glucose in animals, including humans. It is primarily stored in the liver and muscles and is a readily available energy source. Hyaluronic acid is a polysaccharide found in the extracellular matrix of connective tissues, such as skin and cartilage. It is known for its role in lubricating joints and is used in various medical and cosmetic applications. Dextran is a glucose-based polysaccharide produced by bacteria, particularly in the fermentation of sucrose. It has various applications, including as a blood plasma expander and in drug delivery systems. Pectin is a polysaccharide found in the cell walls of fruits, particularly citrus fruits. It is a gelling agent in food processing, particularly in making jams and jellies. Agarose is a polysaccharide extracted from seaweed, primarily used in gel electrophoresis to separate molecules like DNA and proteins.

**Table 2:** Table summarizes some common nanocomposite hydrogel biomaterials derived from macromolecular carbohydrates like cellulose, chitosan, alginate, and their potential applications in tissue engineering, wound healing, drug delivery, and more

Polysaccharide	Nanoparticles/additives	Potential applications
Cellulose-based hydrogels	Nanocellulose, graphene oxide	Tissue engineering (scaffolds)^56^
	Silver nanoparticles	Drug delivery (nanocarriers)^57^
		Wound healing (dressings)^58^
		Cartilage regeneration (3D constructs)^59^
Chitosan-based hydrogels	Chitosan nanoparticles	Wound healing (sponges, films)^60,61^
	Silver nanoparticles	Antibacterial coatings^62^
	Nanoceramic	Bone tissue engineering (scaffolds)^63^
Alginate-based hydrogels	Calcium carbonate, silica	Oral drug delivery (encapsulation)^64^
	Gold nanoparticles	Injectable gels for localized therapy^65^
	Magnetic nanoparticles	3D bioprinting in tissue engineering^66^
Hyaluronic acid-based hydrogels	Hyaluronic acid nanoparticles	Ophthalmic drug delivery (contact lenses)^67^
		Dermal fillers (cosmetic applications)^68^
Collagen-based hydrogels	Collagen nanoparticles	Skin tissue engineering^69^
		Dermal fillers (cosmetic and medical dermatology)^68,70^
Gelatin-based hydrogels	Gelatin nanoparticles	Oral and transdermal drug delivery^71^
		Scaffold development for tissue engineering^72^
Chondroitin sulfate-based hydrogels	Chondroitin sulfate nanoparticles	Cartilage tissue engineering^73^
		Osteoarthritis treatment^74^
Xanthan gum-based hydrogels	Xanthan gum nanoparticles	Controlled release systems^75^
		Gastrointestinal drug delivery^76^
Amylose-based hydrogels	Amylose nanoparticles	Encapsulation of bioactive compounds^77^
		Controlled drug release^78^
Chitin-based hydrogels	Chitin nanoparticles	Wound healing (dressings)^79^
		Scaffold materials for tissue regeneration^80^
Carrageenan-based hydrogels	Silver nanoparticles	Antimicrobial hydrogels^81^
		Drug delivery carriers^82^

Chondroitin sulfate is a sulfated glycosaminoglycan (polysaccharide) found in cartilage and connective tissues. It is commonly used in dietary supplements for joint health. Xanthan gum is a polysaccharide produced through fermentation by the bacterium. It is a thickening and stabilizing agent in various food and industrial applications. Amylose is a linear polysaccharide comprising glucose units linked by α-1,4-glycosidic bonds. It is one of the two components of starch, the other being amylopectin. Chitin is a polysaccharide found in the exoskeletons of arthropods (*e.g.*, insects, crustaceans) and the cell walls of fungi. It is used in various applications, including wound dressings and chitosan production.

## 3. Strategies for nanoparticle incorporation into hydrogel matrices

Incorporating nanoparticles into hydrogel matrices is a versatile approach with applications in various fields, including drug delivery, tissue engineering, and sensor development. The strategies for nanoparticle incorporation into hydrogel matrices depend on the type of nanoparticles, the desired properties of the hydrogel, and the intended application. Here are some strategies to consider:

### 3.1. Physical mixing or stirring

The most straightforward approach involves physically mixing nanoparticles into the hydrogel precursor solution before gelation. This method suits stable nanoparticles that do not agglomerate or settle during gelation.^83^ Care should be taken to achieve uniform dispersion to prevent aggregation. The simplest method for incorporating nanoparticles into hydrogel matrices involves physically mixing the nanoparticles into the hydrogel precursor solution before gelation. This approach suits stable nanoparticles that do not agglomerate or settle during gelation. It is essential to ensure uniform dispersion through controlled stirring, temperature regulation, and pre-dispersion techniques, avoiding air entrainment and considering viscosity. A thorough characterization should be conducted to assess nanoparticle distribution, and compatibility testing should precede the application of the nanoparticle-loaded hydrogel. Various techniques are employed to integrate nanoparticles into hydrogel matrices, enabling the creation of composite materials with enhanced properties. These methods encompass *in situ* synthesis, electrostatic interaction, chemical cross-linking, covalent bonding, freeze-thaw cycling, layer-by-layer assembly, hydrogel nanocomposite formation, ultrasound-assisted dispersion, magnetic field alignment, and microfluidics/emulsion techniques. Each approach facilitates the uniform dispersion of nanoparticles within the hydrogel, contributing to the development of functional materials tailored for applications across diverse fields such as materials science, biomedicine, and nanotechnology.

#### 3.1.1 Stirring/agitation

Using mechanical stirrers, mixers, paddles, *etc.*, to physically move components around and promote interaction. Useful for liquids, pastes, and some solids. This method is advantageous when dealing with hydrogel-particle composite systems, aiding in the uniform distribution of nanoparticles within the hydrogel. Through controlled movement, turbulence, and mixing, stirring promotes interactions between the hydrogel and nanoparticles, ensuring their integration and enhancing compatibility. This approach applies to various hydrogel forms, including liquids and pastes, facilitating the creation of well-dispersed nanoparticle-loaded hydrogel composites suitable for various applications, from biomedicine to materials science. In addition, various mechanical techniques summarized in Table 3 can be employed to ensure the uniform distribution of nanoparticles within hydrogel matrices, enhancing the properties and applications of resulting composite materials.

**Table 3:** Table of the physical mixing and dispersion techniques mentioned, including their advantages, limitations, and advanced features

Technique	Advantages	Limitations	Advanced approaches
Stirring/agitation	Promotes uniform distribution in various hydrogel forms	May not break down tough agglomerates effectively	Use of specialized stirring mechanisms
	Facilitates integration and compatibility	Limited to liquid, paste, and some solid systems	Utilizing flow patterns for improved mixing
High shear mixing	Achieves thorough dispersion and uniformity	Not suitable for highly viscous or solid mixtures	Controlled addition of stabilizers for stability
	Breaks down agglomerates for improved integration	High shear forces may lead to degradation	Sequential addition of components for better mixing
Ultrasonication	Efficiently breaks down particles through cavitation	Limited to liquid dispersions	Frequency and power optimization for better cavitation
	A non-invasive method for improved dispersion	It may require optimization for large-scale processes	Incorporating microfluidics for controlled cavitation
Media/bead mills	Effective for solids and viscous materials	Requires specialized equipment and maintenance	Optimizing bead size and material for efficient grinding
	Promotes uniform dispersion and small particle sizes	Potential for particle contamination	In-line monitoring and control for consistent results
Blending/milling	Reduces particle size for solid hydrogels	Limited to solid and some viscous systems	Inclusion of surfactants to prevent re-agglomeration
	Ensures uniform distribution	Mechanical forces can potentially degrade materials	Utilizing cooling techniques for temperature control
High-pressure homogenization	Achieves submicron particle sizes	Requires specialized equipment and expertise	Parameter optimization for efficient dispersion
	Preserves hydrogel and nanoparticle integrity	Potential for material degradation	In-line monitoring for precise control
Spray drying/chilling	Produces dry powders for various applications	High temperatures may affect temperature-sensitive materials	Modified process conditions for temperature-sensitive materials
	Suitable for liquid hydrogels	Agglomeration during drying	Incorporating stabilizers to prevent agglomeration
	Rapid creation of nanoparticle-loaded powders	The process may not suit all hydrogel formulations	Combining with surface modification for enhanced properties

#### 3.1.2 High shear mixing

Using high-speed mixers, homogenizers, colloid mills, *etc.*, impart high shear forces that break particles/droplets into smaller sizes. It can be used for both liquids and some solids. This technique is valuable for achieving thorough dispersion and uniform distribution of nanoparticles within hydrogel matrices. In the context of hydrogel-nanoparticle composites, high shear mixing helps break up agglomerates and ensures intimate mixing, enhancing the integration and stability of nanoparticles within the hydrogel. This method is suitable for both liquid and paste-like hydrogels, leading to well-dispersed and homogenous nanoparticle-loaded hydrogel systems that can find applications in areas ranging from drug delivery and tissue engineering to advanced functional materials.

#### 3.1.3 Ultrasonication

Applying high-frequency sound waves (ultrasound) to break up particles/droplets through physical cavitation effects. They are commonly used for liquid dispersions. This process involves the formation, growth, and collapse of tiny bubbles in the liquid, generating intense local forces that disrupt agglomerates and enhance dispersion. When applied to hydrogel-nanoparticle systems, ultrasound assists in achieving uniform distribution of nanoparticles within the hydrogel matrix. This method is particularly suitable for liquid hydrogels, facilitating the creation of well-dispersed nanoparticle-loaded hydrogel composites. Ultrasound offers a non-invasive and efficient means of promoting interaction between nanoparticles and hydrogels, leading to enhanced composite properties and potential applications in drug delivery, biomedical engineering, and materials science.

#### 3.1.4 Media/bead mills

Grinding media like zirconia beads impact and break up particles as the mixture is forced between a rotating and stationary surface at high speeds. Effective for solids and highly viscous materials. This technique is highly effective for solid materials and even highly viscous substances. In the context of hydrogel-nanoparticle systems, media mills can facilitate the dispersion and incorporation of nanoparticles within hydrogel matrices. By subjecting the hydrogel-nanoparticle mixture to intense mechanical forces, the milling action helps to break down aggregates and promote uniform distribution of nanoparticles. This method is precious for nanoparticle-loaded hydrogel formulations, aiding in creating well-dispersed and finely homogenized composites with enhanced properties for diverse applications, including drug delivery, tissue engineering, and advanced materials engineering.

#### 3.1.5 Blending/milling

Physically working mixtures through mechanical blending/milling equipment like twin-screw extruders, kneaders, and hammer/ball mills to mix and reduce the particle size of solids. This technique involves physically working the mixture, applying shear forces and mechanical action to break down particles and promote dispersion. In the context of hydrogel-nanoparticle composites, these equipment options can aid in reducing particle size and ensuring uniform distribution of nanoparticles within the hydrogel matrix. This method is particularly well-suited for solid hydrogels or nanoparticle-loaded solid hydrogel formulations, enabling the creation of well-dispersed and finely homogenized composites. By mechanically blending and reducing particle size, these equipment options contribute to developing nanoparticle-loaded hydrogel materials with improved properties for various applications, from biomedical engineering to advanced materials science.

#### 3.1.6 High-pressure homogenization

Forcing liquid dispersions through a narrow gap at extremely high pressures of up to 20 000 psi to break up particles/droplets into the submicron range. This process subjects the mixture to intense shear forces and turbulence, effectively breaking down particles and droplets into the submicron range. In the context of hydrogel-nanoparticle composites, high-pressure homogenization facilitates the dispersion of nanoparticles within the hydrogel matrix, leading to uniform distribution and improved integration. This method is particularly suitable for liquid or gel-like hydrogels, enabling the creation of well-dispersed nanoparticle-loaded hydrogel materials. High-pressure homogenization offers a means to achieve submicron particle sizes and enhance the properties of hydrogel-nanoparticle composites, making it valuable for applications in drug delivery, nanomedicine, and materials engineering.

#### 3.1.7 Spray drying/chilling

Quickly drying or chilling droplets/particles during spray/atomization to promote smaller particle sizes in final powders. In the context of hydrogel-nanoparticle systems, these methods can create nanoparticle-loaded hydrogel powders with enhanced properties. During spray drying, a solution or suspension containing hydrogel and nanoparticles is atomized into fine droplets, which are then exposed to a hot drying gas. The rapid evaporation of the solvent leads to the formation of dry powders, and nanoparticles become incorporated within the hydrogel matrix. On the other hand, spray chilling involves atomizing the mixture into a chilled environment, promoting rapid solidification and particle formation. Both techniques offer means to produce dry nanoparticle-loaded hydrogel powders suitable for various applications, including drug delivery, wound healing, and bioactive materials.

### 3.2. Polymerization strategies

Polymerization methods involve mixing nanomaterial in a neat monomer or a monomer solution. The polymerization process consists of an initiation step followed by a series of polymerization steps, which results in a hybrid between polymer molecules and nanoparticles. This approach is used to develop polymer nanocomposites from nanoparticles. Here are some common strategies for incorporating nanoparticles into hydrogel matrices:

#### 3.2.1 Physical entrapment

Nanoparticles are dispersed in the precursor solution by sonication/stirring, then crosslinked to physically trap the nanoparticles in the polymer mesh. In this approach, nanoparticles are first dispersed within the hydrogel precursor solution through sonication or stirring, ensuring their uniform distribution. Subsequently, the hydrogel precursor is subjected to crosslinking, which forms a three-dimensional polymer network. As crosslinking occurs, the nanoparticles become physically trapped within the polymer mesh, leading to their incorporation into the hydrogel structure. This strategy enables controlled dispersion of nanoparticles and their immobilization within the hydrogel matrix, providing opportunities to tailor the composite's mechanical, chemical, and functional properties for various applications, including drug delivery, tissue engineering, and sensors.

#### 3.2.2 *In situ* synthesis

Nanoparticles are directly synthesized/grown within the hydrogel network during gelation. This method involves the synthesis or growth of nanoparticles directly within the hydrogel network as it undergoes gelation. For instance, silver ions can be reduced *in situ*, leading to the formation and growth of silver nanoparticles within the evolving hydrogel structure. This approach ensures the intimate integration of nanoparticles with the hydrogel and offers precise control over nanoparticle size, distribution, and concentration within the resulting composite. Such *in situ* nanoparticle synthesis within the hydrogel matrix can yield multifunctional materials with enhanced properties, making them suitable for applications like antimicrobial coatings, wound healing, and advanced biomedical technologies.

#### 3.2.3 Covalent bonding

Reactive nanoparticles are functionalized with ligands that can covalently bind to reactive sites on the polymer chains during cross-linking. In this approach, nanoparticles are first modified with ligands that contain reactive functional groups. These ligands are specifically designed to chemically interact with reactive sites on the polymer chains of the hydrogel during the crosslinking process. As the hydrogel precursor undergoes crosslinking, these ligands on the nanoparticles form covalent bonds with the polymer chains, effectively anchoring the nanoparticles within the hydrogel matrix. This strategy allows for precise control over the incorporation of nanoparticles and can lead to a well-integrated composite material with enhanced mechanical, chemical, and functional properties suitable for various applications. Li *et al.*^84^ investigated a new type of hydrogel actuator that can undergo rapid snapping motion under an electric field. The hydrogel is a polyelectrolyte gel disk embedded in a neutral gel frame. Due to swelling mismatch, the polyelectrolyte gel buckles out-of-plane into a dome shape with bistability. A novel approach is introduced to develop polyampholyte (PA) hydrogels with a unique size-mechanical property relationship, exhibiting a swelling yet strengthening behavior. The method involves dialyzing a dynamic PA hydrogel, rich in ionic bonds, successively in ZrOCl_2_ solutions (Step-I) and deionized water (Step-II). The hydrogel networks are reorganized through specific Zr^4+^ ions and the PA network structure, resulting in a continuous increase in sample size and mechanical performance over several months in Step-I, while Step-II only requires a few days. The multiphase microstructures of the hydrogels are found to be influenced by the dialysis time in Step-I and the corresponding ZrOCl_2_ concentration, leading to varied mechanical enhancements. Despite significant swelling, the optimized hydrogel achieves a Young's modulus of 39.2 MPa and tensile strength of 3.7 MPa—302 and 5.5 times those of the original PA gel, respectively. This innovative strategy paves the way for the fabrication of hydrogels that combine substantial swelling with enhanced mechanical strength, surpassing many existing high-performance hydrogels. When an electric field is applied, mobile ions inside the polyelectrolyte gel migrate towards the electrodes. This creates an osmotic pressure gradient that drives the migration of water molecules and the bending of the gel. However, the surrounding neutral gel constrains the bending motion, causing elastic energy to accumulate until a threshold is reached and the gel suddenly snaps through.^85^ Y. Huang, *et al.* represent a simple method of fabricating strong and tough polyampholyte hydrogels using ionic and metal–ligand bonds. Conventional hydrogels are mechanically weak due to their high water content. The researchers propose a secondary equilibrium approach to enhance the mechanical properties of polyampholyte hydrogels. First, the original polyampholyte gels are soaked in multivalent metal ion solutions to reach a swelling equilibrium. This destroys most of the ionic bonds in the gel network and allows for reorganization. Then, the gels are transferred to deionized water to remove excess metal ions and counter ions. This achieves a new equilibrium state where the gel network is constructed by both ionic and metal–ligand bonds, synergistically reinforcing the hydrogel. Using this approach with ferric chloride solution, the researchers enhanced Young's modulus, tensile strength, and work of tension of the polyampholyte gels by up to 200%, 192%, and 356%, respectively. The concentration of metal ions in the soaking solution affected the degree of enhancement. Optimally, 0.7 molar ferric chloride solution gave the best results. The approach was also generalizable using different polyampholyte gel systems and metal ions like aluminum, zinc and calcium. The resultant hydrogels also exhibited stable ion conductivity even after equilibrating in water, making them suitable as stretchable strain sensors.^86^

#### 3.2.4 Electrostatic interactions

Oppositely charged nanoparticles and polymers self-assemble through coulombic forces. An example is amine-modified nanoparticles in alginate hydrogel. When oppositely charged nanoparticles and polymers are combined, they can spontaneously self-assemble due to coulombic (electrostatic) interactions between the charges. For instance, amine-modified nanoparticles, which carry a positive charge, can interact with negatively charged groups on polymers such as alginate.^87^ This electrostatic attraction leads to a stable composite, where nanoparticles become embedded within the polymer matrix. This self-assembly approach offers a straightforward way to incorporate nanoparticles into hydrogels, providing control over distribution and loading while utilizing the attractive forces between charged entities. This technique can be harnessed for various applications ranging from drug delivery and tissue engineering to responsive materials and nanocomposites.

#### 3.2.5 Hydrophobic interactions

Hydrophobic nanoparticles insert into hydrophobic domains/pockets within amphiphilic polymer chains. When hydrophobic nanoparticles come into contact with amphiphilic polymers, which possess both hydrophilic and hydrophobic regions, a phenomenon known as “hydrophobic interaction” can occur. Hydrophobic portions of the amphiphilic polymer chains tend to cluster together to minimize contact with water, creating hydrophobic domains or pockets. Due to their similar nature, hydrophobic nanoparticles can insert themselves into these hydrophobic regions of the polymer chains, seeking to minimize their exposure to the surrounding aqueous environment. This interaction can lead to the encapsulation or incorporation of nanoparticles within the polymer matrix, providing a means to engineer composite materials with controlled properties and functionalities for various applications, including drug delivery, nanocomposites, and controlled release systems.

#### 3.2.6 Host–guest interactions

Supramolecular host units on nanoparticles non-covalently bind guest units on polymeric backbones. In this scenario, supramolecular host units on nanoparticles and guest units on polymeric chains engage in non-covalent binding interactions. These interactions, including hydrogen bonding, hydrophobic interactions, and π–π stacking, create reversible and dynamic connections between the nanoparticles and the polymer. This supramolecular assembly approach allows for the design of functional nanocomposites with tunable properties, where the interactions between the host and guest units enable controlled incorporation, release, or arrangement of nanoparticles within the polymeric matrix. This strategy holds promise for drug delivery, responsive materials, and nanotechnology applications.

#### 3.2.7 Layer-by-layer assembly

Alternate deposition of nanoparticles and polyelectrolytes on a substrate through electrostatic layering. In this technique, charged nanoparticles and polyelectrolytes are sequentially deposited onto a substrate through electrostatic interactions. The process involves the attraction between oppositely charged species, forming alternating layers of nanoparticles and polyelectrolytes on the substrate surface. Each deposition cycle involves adsorption of the charged species, followed by rinsing to remove any unbound material. This layer-by-layer assembly method allows precise control over the thickness and composition of the deposited layers, facilitating the creation of multilayered nanocomposite coatings or films. The approach has applications in surface modification, controlled drug release, sensors, and nanotechnology.

#### 3.2.8 Molecular imprinting

Functional monomers and nanoparticles are copolymerized to create recognition sites for specific nanoparticles. Molecular imprinting involves the creation of specific binding sites (recognition sites) within a polymer matrix by copolymerizing functional monomers and often nanoparticles around a template molecule. The template is then removed, leaving behind voids or imprints that are complementary in shape and functionality to the template. These imprints enable the selective capture and recognition of the target molecule or nanoparticles upon subsequent use. The technique offers a way to engineer particular and selective binding materials for applications such as sensors, separation, and drug delivery.

#### 3.2.9 Microfluidic assembly

Controlled mixing of nanoparticle and polymer solutions through microchannels leads to uniform dispersion. Microfluidic assembly involves precisely manipulating fluids, such as nanoparticle and polymer solutions, within microscale channels to achieve controlled mixing and dispersion by carefully designing the geometry of the microchannels and the flow rates of the solutions, intimate and efficient mixing occurs, leading to uniform dispersion of nanoparticles within the polymer solution. This approach allows fine-tuned control over the nanoparticle-polymer interaction, resulting in well-defined and homogenous composite materials. Microfluidic assembly is valuable for creating advanced materials with tailored properties for applications ranging from drug delivery and diagnostics to nanotechnology and tissue engineering.

The choice of strategy depends on various factors, including the nature of the nanoparticles, the hydrogel material, desired properties, and the intended application. Combining multiple techniques can often provide better control over the incorporation process and enhance composite materials. This approach allows for synergistic effects and optimization of the composite's properties by leveraging the strengths of different methods. Careful consideration and experimentation are crucial to selecting and combining techniques to achieve the desired performance and functionality of the nanoparticle-loaded hydrogel for specific applications.

### 3.3. Surface modification approaches for enhanced compatibility

Surface modification approaches play a pivotal role in enhancing the compatibility and performance of metal hydrogel nanocomposites in various applications.^88^ Metal hydrogel nanocomposites combine the unique properties of metals with the versatility of hydrogels, opening up a realm of possibilities in fields such as biomedical engineering, catalysis, and environmental remediation. However, achieving optimal interactions between the metal nanoparticles and the hydrogel matrix is essential to unlocking their full potential. Surface modification techniques offer tailored solutions to address challenges such as nanoparticle agglomeration, limited dispersion, and suboptimal binding within the hydrogel network. One common surface modification strategy involves functionalizing the metal nanoparticles with specific ligands or polymers with an affinity for the hydrogel matrix.^89^ This approach enhances the compatibility between the hydrogel and the metal nanoparticles and provides additional functional groups for further customization. By carefully selecting the type and density of functional groups, researchers can achieve improved dispersion, stability, and interaction between the components, leading to enhanced overall performance. Another promising surface modification approach is the creation of intermediate layers or coatings on the metal nanoparticles before their incorporation into the hydrogel matrix. These intermediate layers can bridge the metal nanoparticles and the hydrogel, facilitating stronger and more stable interactions. For instance, polymer coatings can provide a protective shell around the metal nanoparticles, preventing aggregation and enhancing their dispersibility within the hydrogel.

Furthermore, surface modification techniques can be tailored to optimize specific properties of the metal hydrogel nanocomposites. For example, in biomedical applications, the biofunctionalization of metal nanoparticles can facilitate targeted interactions with cells or biomolecules, promoting biocompatibility and cellular adhesion. In catalysis, surface modification can create catalytic active sites on the metal nanoparticles, enhancing their efficiency in driving chemical reactions.

In conclusion, surface modification approaches offer versatile tools to enhance the compatibility and performance of metal hydrogel nanocomposites. These strategies enable precise control over interactions at the nanoscale and provide opportunities for customization to meet the specific requirements of diverse applications. As research in this field advances, innovative surface modification techniques continue to contribute to developing highly functional and practical metal hydrogel nanocomposites with broad-reaching implications. Here are some common surface modification approaches used to enhance the compatibility of nanoparticles for incorporation into hydrogel matrices:

#### 3.3.1 PEGylation

Conjugating polyethylene glycol (PEG) chains onto the nanoparticle surface improves solubility biocompatibility and reduces non-specific interactions. PEGylation involves attaching polyethylene glycol (PEG) chains onto the surface of nanoparticles, resulting in a PEGylated nanoparticle. This modification offers several advantages, including enhanced solubility in aqueous solutions, improved biocompatibility, and reduced non-specific interactions with biological components. The hydrophilic PEG chains create a protective “shield” around the nanoparticle, minimizing its recognition by the immune system and reducing protein adsorption.^90^ This strategy is widely used to improve the pharmacokinetics and biodistribution of nanoparticles, making them more suitable for applications in drug delivery, imaging, and diagnostics, among others.^91^ Research suggests that a minimum molecular weight (MW) of 2000 or higher is required for PEG chains on the nanoparticle surface to avoid the immune system's MPS (mononuclear phagocyte system) and increase circulation half-life. Shorter PEG chains are less flexible and may not provide the desired protective effect. Increasing the molecular weight of PEG chains beyond 2000 may further enhance the blood circulation half-life of PEGylated particles. This could be due to the increased chain flexibility of higher MW PEG polymers, which may contribute to improved evasion of immune responses.

**Fig. 2:**
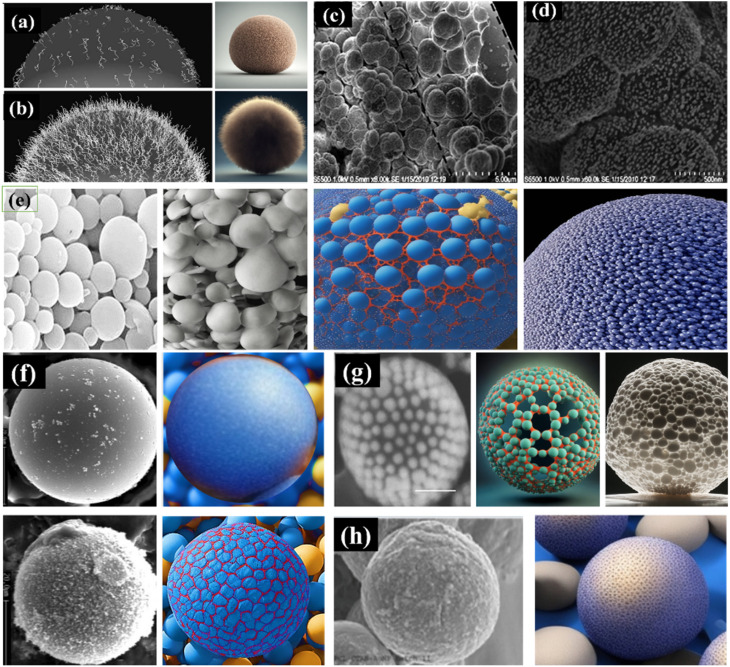
Figure shows surface modification approaches for enhanced compatibility of metal nanocomposite hydrogel (a) brush and (b) mushroom structure of PEG chains on the surface of nanoparticles, (c) and (d) show the attachment of AuNPs to the monolith surface began with the amination of the monolith surface, (e) gold nanoparticles coated by two later of polyacrylic acid and polyacrylamide, (f) the UF nanoparticles before and after silanization process, (g) gold nanoparticles on the grafting amphiphilic block copolymers BCPs (PEO_45_-*b*-PS_455_-SH), (h) poly(ε-caprolactone) (PCL) microparticles have been engineered to encapsulate colloidal gold along with RGD peptide. Copyright with permission.^91,94–97^

The density of PEG chains on the nanoparticle surface and their conformation significantly affect the stealth characteristics of the particles. At low surface coverage, PEG chains exhibit a “mushroom” configuration, which allows for a more extensive range of motion and closer proximity to the nanoparticle surface. However, this configuration might lead to gaps in the PEG layer where opsonin proteins (which mark foreign particles for removal) can bind. High surface coverage of PEG chains leads to a “brush” configuration, where the chains are extended away from the nanoparticle surface. While this ensures better particle coverage, it can also limit the mobility of the PEG chains and reduce their steric hindrance properties. Steric hindrance refers to the physical obstruction of interactions between the nanoparticle surface and surrounding biological molecules, which aids in evading recognition by the immune system. The provided schematic diagram (Fig. 2a and b) illustrates these two configurations, the “mushroom” and “brush” – of PEG chains on the surface of nanoparticles.^91^

#### 3.3.2 Amination

Using amines as surface modifiers provides cationic surface charge to facilitate attachment to anionic polymer chains through electrostatic interactions.^92^ This cationic charge facilitates electrostatic interactions with anionic polymer chains, leading to binding solids and attachment between the nanoparticles and the polymer matrix.^93^ This approach improves compatibility and stability in cases where the polymer is negatively charged. The electrostatic attraction between the cationic nanoparticles and anionic polymer chains promotes effective incorporation and dispersion, making amination a valuable technique for engineering nanoparticle–polymer composites in various applications such as drug delivery, tissue engineering, and nanocomposite materials. Fig. 2c and d show that the attachment of AuNPs to the monolith surface began with the amination of the monolith surface. For instance, as outlined by,^94^ modifying the polymer monolith with gold nanoparticles (AuNPs) and aminated groups involves a strategic process to enhance the monolith's surface properties and functionality. Based on a protocol with modifications, the method facilitates the covalent attachment of AuNPs onto the monolith surface through amino groups. The process can be summarized as follows. Grafted polymer chains of vinyl azlactone on the monolith surface are reacted with ethylenediamine. This chemical reaction introduces amino groups onto the monolith, creating specific sites for subsequent attachment. Now featuring amino groups, the monolith is exposed to 20 nm citrate-stabilized AuNPs. These AuNPs are prepared through the citrate reduction method. The amino groups on the monolith surface form multi-point interactions with the AuNPs, leading to their covalent attachment. The uniformity of AuNP coverage on the monolith is assessed using Field Emission Scanning Electron Microscopy (FE-SEM). This analysis reveals the distribution and density of attached AuNPs across the monolith surface. The successful attachment of AuNPs results in an increased surface area of the monolith due to the presence of the nanoparticles. This enhancement contributes to improved surface properties and functionality. The modification process leverages the unique properties of AuNPs and aminated groups to create a functionalized monolith with enhanced capabilities. The covalent attachment of AuNPs through amino groups introduces nanoscale features and interactions, enriching the monolith's surface for potential applications in various fields.

#### 3.3.3 Carboxylation

Carboxyl groups on the nanoparticle surface can form ionic and covalent bonds with cationic or cross-linkable groups on polymers. Carboxylation involves introducing carboxyl groups onto nanoparticle surfaces, enabling ionic interactions with cationic groups on polymers, and facilitating covalent bonds through cross-linking or anchoring during polymerization. This surface modification strategy enhances compatibility and incorporation between nanoparticles and polymers, leading to well-integrated and stable composites with improved properties, making them valuable for applications such as drug delivery, sensing, and nanomaterial engineering. Fig. 2e shows gold nanoparticles coated by polyacrylic acid and then coated by polyacrylamide.

#### 3.3.4 Silanization

Silane coupling agents can covalently attach to inorganic nanoparticles and contain functional groups for bonding with polymers. These coupling agents contain reactive functional groups, such as silanol (Si–OH) groups, that can chemically bond with the nanoparticle surface. These functionalized nanoparticles can also interact with polymers through the same functional groups, establishing covalent bonds and enhancing compatibility. Silanization offers a versatile approach for tailoring nanoparticle–polymer interactions, leading to composite materials with improved stability, dispersion, and targeted properties for applications ranging from coatings and composites to biomedical devices and sensors. Fig. 2f illustrates scanning electron microscopy (SEM) images of UF (urea–formaldehyde) microcapsules before and after undergoing functionalization through the use of a silane-coupling agent.^95^ The findings from the images provide clear evidence of the binding of silane molecules onto the surface of the functionalized UF microcapsules. Notably, a noticeable observation is that the surface texture of the functionalized microcapsules appears to be more uneven or roughened than the original microcapsules. Additionally, a thin layer has formed, effectively covering the surfaces of the microcapsules that underwent functionalization. These visual comparisons between the pre-functionalized and post-functionalized microcapsules provide insights into the successful attachment of the silane molecules and the resulting alteration of surface properties.

#### 3.3.5 Polymer grafting

Short polymer chains like poly(acrylic acid) and poly(vinyl alcohol) are grafted to provide functional groups and steric hindrance. These grafted polymer chains can provide functional groups that contribute to specific interactions (*e.g.*, hydrogen bonding) and offer steric hindrance, preventing agglomeration and enhancing stability. This technique modifies the surface properties of substrates or nanoparticles, improving compatibility with other materials like hydrogels. The grafted polymers introduce desired functionalities and act as spacers, reducing intermolecular interactions and improving dispersibility. This strategy finds applications in diverse fields, including drug delivery, surface coatings, and nanocomposite fabrication. The grafting amphiphilic block copolymers BCPs (PEO_45_-*b*-PS_455_-SH) are attached to the surface of gold nanoparticles (AuNPs) through covalent bonds known as Au–S bonds Fig. 2g. This attachment is achieved using a method called the solution ligand exchange approach. In this process, the ligands on the surface of the AuNPs are exchanged with the amphiphilic BCPs through chemical reactions, resulting in the covalent bonding of the BCPs to the AuNPs. This functionalization strategy enables the modification of AuNPs with the amphiphilic BCPs, imparting specific properties and behaviors to the resulting nanocomposite material^96^

#### 3.3.6 Protein/peptide conjugation

Biospecific ligands like RGD peptides improve cellular interaction and compatibility. These ligands enhance the interaction between the nanoparticles and cells by promoting specific recognition and binding to cellular receptors. RGD peptides, for example, facilitate cell adhesion and can improve biocompatibility by mimicking natural extracellular matrix components. This strategy leverages the inherent biological functions of the ligands to enhance cellular targeting, internalization, and overall compatibility. Protein or peptide conjugation is a powerful approach for developing targeted drug delivery systems, tissue engineering scaffolds, and other biomedical applications where precise cellular interactions are crucial for success. Fig. 2g shows poly(ε-caprolactone) (PCL) microparticles have been engineered to encapsulate colloidal gold along with RGD peptide, a specific molecular sequence that exhibits an affinity for and binds to colon tumors that overexpress the integrin receptor. This targeted design aims to enhance the delivery of therapeutic or imaging agents to colon tumors, taking advantage of the integrin receptor's presence on the tumor cells.^97^ The RGD peptide serves as a homing mechanism, guiding the PCL microparticles loaded with colloidal gold to specifically accumulate at colon tumors where integrin receptors are abundantly expressed. This approach holds the potential for more effective and precise diagnostics or treatment strategies for colon cancer.^98^

#### 3.3.7 Zwitterionization

Zwitterionic groups like phosphorylcholine confer stealth properties and prevent nonspecific protein adsorption. Zwitterionic molecules have positively and negatively charged groups within the same molecule, creating a neutral overall charge. This unique property imparts excellent resistance to nonspecific protein adsorption and cell adhesion, making zwitterionic surfaces “protein-resistant” or “stealthy.” By minimizing interactions with proteins and cells, zwitterionic coatings enhance biocompatibility, reduce immune responses, and prolong the circulation time of nanoparticles, making them highly valuable for applications in drug delivery, medical implants, and biosensors.

#### 3.3.8 Dendrimer coating

Dendrimers encapsulate nanoparticles and present desired surface functionality. Dendrimers, highly branched and well-defined macromolecules, can be designed to encapsulate nanoparticles within their core. This encapsulation protects the nanoparticles and can control their release behavior. Additionally, dendrimers' outer surface can be functionalized with specific molecules to confer desired surface properties, such as targeting ligands or stealth characteristics. This unique combination of core encapsulation and surface modification makes dendrimers a versatile platform for developing advanced nanocomposite materials with applications in drug delivery, imaging, and other biomedical fields.

The objective is to incorporate functional chemical moieties onto the nanoparticle surface to enhance compatibility, ensure steric or electrostatic stabilization, and enhance hydrophilicity and biocompatibility. Introducing these modifications allows the nanoparticles to integrate more effectively within hydrogel systems. The functional groups promote specific interactions with the hydrogel matrix, preventing agglomeration and enhancing stability. The altered surface chemistry makes the nanoparticles more hydrophilic, reducing non-specific interactions and making them more compatible with the aqueous environment of hydrogels. This approach aims to optimize the synergistic integration of nanoparticles and hydrogels, yielding composite materials suitable for various applications, from drug delivery and tissue engineering to advanced biomaterials.

## 4. Tailoring hydrogel material properties through nanoparticle selection and loading

Nanoparticles' size, shape, and surface chemistry impact their dispersion and interactions in hydrogels. Smaller, high-aspect-ratio particles often reinforce better. Optimizing loading levels is crucial; too low yields minimal effects, while excessive loading leads to aggregation and degradation. A percolation threshold must be achieved for enhancements. In metal hydrogel nanocomposites, reaching the percolation threshold is a pivotal goal. Below this threshold, the dispersed metal nanoparticles may not effectively influence the properties of the composite,^99^ and any enhancements observed could be limited or negligible. However, a transformative change occurs as the concentration of metal nanoparticles gradually increases and approaches the percolation threshold.

At or beyond the percolation threshold, the metal nanoparticles form a continuous network or cluster within the hydrogel matrix. This network allows for efficient electron transport, improved mechanical reinforcement, enhanced electrical conductivity, and altered optical properties. As a result, the nanocomposite experiences a significant boost in its desired functionalities, making it suitable for various applications, from sensing and catalysis to tissue engineering and beyond. In percolation theory, researchers often strive to characterize and quantify the specific concentration at which the percolation threshold occurs. This may involve experimental investigations, computational simulations, and advanced analytical techniques. By precisely understanding and controlling the percolation threshold, scientists and engineers can tailor the composition and structure of metal hydrogel nanocomposites to achieve the desired enhancements in properties, paving the way for innovative and high-performance materials. Uniform dispersion *via* methods like *in situ* synthesis during gelation is vital. Interfacial interactions between nanoparticles and hydrogel polymer chains affect stress transfer and enhancement degree. Further crosslinking or polymer-particle grafting may be necessary for integration and stable performance. The resulting nanocomposite hydrogels exhibit improved strength, conductivity, and stimuli-responsiveness, tailored for applications like biomedicine or sensors.

### 4.1. Influence of nanoparticle size, shape, and composition

Nanoparticles profoundly impact hydrogel properties, and their size, shape, and composition intricacies govern this influence. Each parameter is distinctive in dictating how nanoparticles disperse within hydrogels and interact with the polymer matrix, thereby sculpting the resulting material's characteristics. Size dramatically alters the distribution and integration of nanoparticles within hydrogels. Smaller nanoparticles offer higher surface area-to-volume ratios, bolstering their dispersion and potential for interlocking within the hydrogel network. Such intimate interaction can lead to enhanced reinforcement and property modulation. Shape introduces an additional layer of complexity. High-aspect-ratio nanoparticles, like nanotubes or nanowires, often exhibit superior reinforcement capabilities due to their elongated geometry, enabling efficient stress transfer within the hydrogel.

Conversely, spherical nanoparticles may provide isotropic property enhancements. The intrinsic composition of nanoparticles profoundly influences hydrogel behavior. Metallic, polymeric, magnetic, or biologically active materials bestow unique attributes onto the nanocomposite. This composition-dependent synergy with the hydrogel matrix endows tailored properties, ranging from enhanced mechanical strength to bioactivity. Fine-tuning nanoparticle size, shape, and composition is paramount to achieving desired property enhancements. An optimal combination can induce a percolation threshold—an ideal nanoparticle concentration at which significant improvements manifest. Achieving uniform dispersion, possibly through *in situ* synthesis during hydrogel formation, ensures consistent and efficient property modulation. Interactions at the nanoparticle–hydrogel interface influence stress transfer and the extent of property enhancement. Tailoring surface chemistry or employing grafting techniques can enhance compatibility, strengthening the interplay between nanoparticles and polymer chains.

### 4.2. Tuning mechanical strength and flexibility

The mechanical properties of hydrogels play a pivotal role in their applicability across diverse fields. Tailoring these properties-specifically strength and flexibility—through nanoparticle integration offers a potent avenue for enhancing hydrogel functionality, durability, and suitability for various applications. Incorporating nanoparticles, carefully chosen for their size, shape, and compatibility, can significantly reinforce hydrogel mechanical strength. Nanoparticles with high aspect ratios or exceptional stiffness can act as structural reinforcements, distributing stress and enhancing overall material toughness. Achieving the right balance of nanoparticle loading is a delicate endeavor. Limited reinforcement results from insufficient nanoparticles and mechanical characteristics might be impaired by agglomeration and heavy loading.^100^ Precise loading levels, often guided by percolation thresholds, must be reached to unlock significant enhancements. The interactions between nanoparticles and hydrogel polymer chains at the interface are crucial. Effective stress transfer and load distribution rely on interfacial solid connections. Surface modifications or functionalization can bolster interfacial interactions, augmenting mechanical performance. Strategic crosslinking or altering the hydrogel network structure can further optimize mechanical attributes. Combining these design approaches with nanoparticle integration allows for fine-tuning mechanical strength and flexibility, tailoring hydrogels to withstand specific stresses and strains. Enhancing mechanical properties not only fortifies hydrogels but also expands their applications. Nanoparticle-reinforced hydrogels find utility in tissue engineering scaffolds, wound dressings, and drug delivery carriers, where mechanical resilience is pivotal for successful outcomes. Balancing mechanical strength with flexibility is crucial for hydrogels that mimic biological tissues or accommodate dynamic environments. Nanoparticles can offer controlled flexibility, allowing hydrogels to respond to external stimuli or physiological changes while maintaining structural integrity.

In summation, nanoparticle-induced mechanical reinforcement presents a transformative strategy for tuning the strength and flexibility of hydrogels. Integrating nanoparticles with careful design and interfacial engineering enriches the mechanical toolkit for crafting hydrogels with advanced properties catered to specific demands.

### 4.3. Balancing drug loading and release kinetics

The controlled delivery of therapeutic agents from hydrogels is a cornerstone of drug delivery systems. Achieving equilibrium between drug loading and release kinetics is pivotal for optimizing therapeutic efficacy, minimizing side effects, and tailoring hydrogels for targeted applications. Nanoparticles offer a platform to load drugs within hydrogels efficiently. Drug loading can be optimized by harnessing the nanoparticles' high surface area and tunable interactions. The selection of nanoparticle type and surface modifications influences drug-nanoparticle affinity and capacity. Nanoparticles profoundly impact drug release kinetics. Nanoparticle size, porosity, and composition influence diffusion pathways and release rates. Tailoring these parameters enables fine-tuning drug release profiles, ranging from sustained to rapid, on-demand release. Combining nanoparticles with hydrogels creates a synergistic system where nanoparticles act as reservoirs or carriers, modulating drug release. The interplay between nanoparticle properties and hydrogel network swelling profoundly influences drug diffusion and release. Nanoparticles can confer responsiveness to external stimuli, triggering controlled drug release. Temperature, pH, light, or magnetic fields can manipulate release kinetics, enabling precise drug delivery in response to specific cues. Achieving an optimal balance between drug loading and release kinetics is a delicate endeavor. Higher drug loading may lead to burst release, while lower loading may result in insufficient therapeutic levels. Nanoparticle integration facilitates a rational approach to achieving desired release profiles. Nanoparticle-loaded hydrogels find applications in diverse fields, including wound healing, cancer therapy, and regenerative medicine. Tailoring drug release kinetics allows hydrogels to be customized for specific therapeutic requirements and patient needs. The ability to precisely control drug release kinetics empowers the realization of personalized medicine. Nanoparticle-incorporated hydrogels provide a versatile platform for designing patient-specific drug delivery systems, enhancing treatment outcomes, and minimizing adverse effects. Nanoparticle-based strategies offer a dynamic avenue for advancing drug delivery from hydrogels. The synergy between nanoparticle characteristics and hydrogel properties is poised to revolutionize therapeutic interventions, heralding a new era of targeted and controlled drug release.

In summary, nanoparticle integration within hydrogels heralds a paradigm shift in optimizing drug loading and release kinetics. This approach can revolutionize drug delivery, enabling precise, tailored, and responsive therapeutic interventions.

## 5. Exploring the synergy between nanotechnology and hydrogel chemistry

The convergence of nanotechnology and hydrogel chemistry represents a transformative frontier where innovative materials emerge by leveraging the distinctive attributes of both domains. This synergistic collaboration unlocks many possibilities, revolutionizing material design, functionality, and application versatility. Nanotechnology seamlessly integrates with hydrogel chemistry, augmenting material properties beyond traditional boundaries. Nanoparticles, chosen for size, composition, and surface characteristics, dynamically interact with hydrogel networks, leading to enhanced mechanical strength, controlled drug delivery, or responsive behavior. By incorporating nanoparticles, hydrogel structures can be tailored at the nanoscale. Reinforcement with high-aspect-ratio nanoparticles endows hydrogels with impressive mechanical robustness, while the precise arrangement of nanoparticles can influence hydrogel porosity and permeability. Nanoparticles introduce responsive attributes to hydrogels, transforming them into intelligent materials. Swelling behavior, drug release kinetics, or mechanical properties can be tuned in response to external stimuli, enabling applications like intelligent drug delivery systems or adaptable tissue scaffolds. Nanoparticles functionalized with biomolecules seamlessly integrate into hydrogels, fostering biocompatibility and bioactivity. The resulting hybrid materials facilitate cell adhesion, tissue regeneration, and bioactive molecule delivery, extending hydrogel applications in regenerative medicine and tissue engineering. Nanotechnology empowers precise control over drug loading, release kinetics, and targeting within hydrogels. Nanoparticles act as reservoirs or carriers, enabling on-demand or sustained drug release, revolutionizing therapeutic interventions, and minimizing side effects. Nanoparticles embedded in hydrogels create platforms for real-time sensing and monitoring. This synergy spawns biosensors and diagnostic devices that detect specific analytes or changes in physiological conditions, presenting groundbreaking avenues in healthcare and diagnostics.

The symbiotic relationship between nanotechnology and hydrogel chemistry holds immense promise. It underpins the creation of novel materials with multifaceted attributes, bridging the gap between synthetic and biological systems. From personalized medicine to wearable technologies, this synergy catalyzes unprecedented advancements. While this synergy offers boundless opportunities, challenges like nanoparticle dispersion, biocompatibility, and long-term stability require thoughtful consideration. Successful navigation of these challenges yields materials that redefine the boundaries of possibility, forging new frontiers in science and engineering.

In summary, the harmonious fusion of nanotechnology and hydrogel chemistry is a testament to the remarkable potential of interdisciplinary collaboration. This synergy opens avenues for pioneering materials, with tailored properties poised to revolutionize healthcare, biotechnology, and beyond industries.

### 5.1. Nanoparticle–hydrogel interactions at the molecular level

At the nexus of nanotechnology and hydrogel chemistry lies a captivating realm of molecular interactions that intricately shape material behavior and functionality. These interactions at the nanoscale orchestrate the synergy between nanoparticles and hydrogels, dictating properties and applications. The interplay begins with surface chemistry—nanoparticles are endowed with functional groups that dictate their affinity to hydrogel polymer chains. Hydrogen bonding, electrostatic attractions, and covalent linkages establish a binding between nanoparticles and hydrogels, influencing dispersion and structural integration. Nanoparticles intricately influence hydrogel swelling behavior and conformational changes. By affecting the water uptake and polymer network dynamics, nanoparticles govern hydrogel responsiveness to external stimuli, paving the way for intelligent materials that adapt to changing environments. Nanoparticles bolster mechanical properties by actively transferring stress within the hydrogel matrix. Surface-functionalized nanoparticles form robust interfacial connections with polymer chains, creating a composite system with enhanced load-bearing capacities, broadening applications in tissue engineering and beyond. The molecular interactions enable nanoparticles to confer responsiveness to hydrogels. pH, temperature, or external fields prompt changes in nanoparticle–hydrogel interactions, regulating material properties. Such responsive modulation has far-reaching implications, from controlled drug delivery to environmental sensing. Molecular-level interactions intricately govern drug loading and release kinetics. Hydrophobic interactions, hydrogen bonding, or specific receptor-ligand binding dictate drug incorporation and release from nanoparticles within the hydrogel network, unlocking tailored therapeutic strategies. The strength of nanoparticle–hydrogel interactions influences long-term stability.

**Fig. 3:**
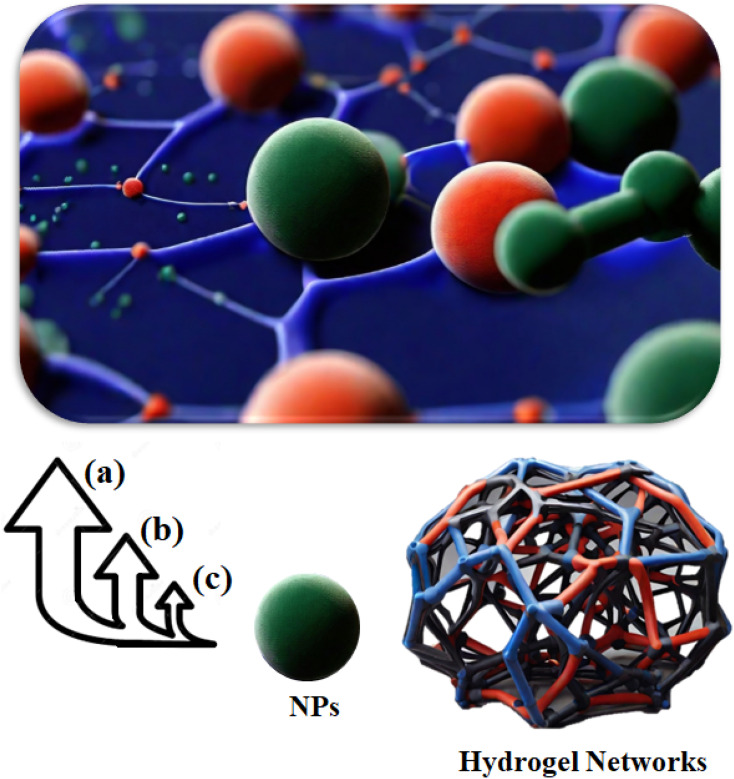
Figure illustrates various approaches for preparing nanoparticle–hydrogel composites.

Effective interactions mitigate particle agglomeration, ensuring sustained enhancement of hydrogel properties over time. Optimized interactions extend material longevity, pivotal for applications like implants or long-term drug delivery. Understanding and controlling these molecular interactions is an intricate challenge and a frontier of exploration. Tailoring surface chemistry, optimizing binding mechanisms, and deciphering nanoparticle-induced structural changes are critical steps toward harnessing the full potential of this molecular synergy. By unraveling the secrets of nanoparticle–hydrogel interactions, we can design materials with unprecedented precision tailored for specific functions and applications, from personalized medicine to advanced electronics. Fig. 3 shows that incorporating nanoparticles (NPs) into hydrogels can be achieved through various routes. NPs can be added to (a) pre-formed hydrogels; (b) polymer solutions that are subsequently gelled, either utilizing the NPs as cross-linkers or independently of their presence; or (c) monomer solutions before co-cross-linking polymerization in the presence of the NPs. Additionally, it is possible to grow the NPs from precursors incorporated into the polymer network using strategies (a) and (b). These diverse approaches provide flexibility in incorporating NPs into hydrogel matrices, offering a range of methods for tailored composite material synthesis.

### 5.2. Manipulating crosslinking density for controlled swelling

In the intricate realm where nanotechnology converges with hydrogel chemistry, the manipulation of crosslinking density emerges as a powerful tool for tailoring the swelling behavior of hydrogels. This fine-tuned control over swelling responses offers a gateway to designing intelligent materials with applications ranging from controlled drug delivery to responsive biomimetic systems. Crosslinking density profoundly governs hydrogel swelling. The degree of swelling can be precisely regulated by strategically varying the number and strength of crosslinks between polymer chains. This manipulation enables hydrogels to exhibit controlled responsiveness to external stimuli. Nanoparticles introduce an added dimension to crosslinking control. The interplay between nanoparticle incorporation and crosslinking dictates the hydrogel's behavior in response to factors like pH, temperature, or specific ions. This responsiveness underpins the development of intelligent hydrogels with adaptable swelling characteristics. Crosslinking density plays a pivotal role in modulating drug release kinetics. By judiciously adjusting the degree of crosslinking, the diffusion pathways for drug molecules within the hydrogel can be regulated. This controlled release provides a mechanism for optimizing therapeutic outcomes. Nanoparticles can enhance crosslinking by facilitating additional interaction points. Functionalized nanoparticles introduce sites for crosslinking or alter the polymer network structure, influencing both the hydrogel's mechanical properties and its swelling behavior. Crosslinking density impacts not only swelling but also hydrogel stability and durability. Nanoparticle-induced crosslinking modifications can fortify the hydrogel structure, contributing to prolonged material integrity and enhanced performance over time. The intricate interplay between crosslinking and nanoparticle interactions allows for creation of biomimetic hydrogel systems. These materials can replicate the dynamic swelling behavior of natural tissues, enabling applications in tissue engineering, wound healing, and beyond. Tailoring crosslinking density in the presence of nanoparticles poses challenges that warrant exploration. Balancing structural modifications, surface functionalization, and swelling behavior necessitates a deep understanding nanoscale interactions within the hydrogel network. The synergy between nanoparticle-enhanced crosslinking and hydrogel swelling presents a realm ripe for innovation. With the ability to engineer hydrogels with tunable and predictable swelling responses, this approach can potentially revolutionize fields such as drug delivery, sensors, and artificial tissues.

In conclusion, the artful manipulation of crosslinking density in the presence of nanoparticles heralds a new era of hydrogel design. This dynamic interplay allows for the creation materials that elegantly respond to external cues, offering unprecedented control over swelling behavior and paving the way for multifunctional, responsive, and adaptable hydrogel systems.

### 5.3. Enhancing biofunctionalization for specific applications

The synergy between nanotechnology and hydrogel chemistry extends its reach to biofunctionalization, where the precise integration of biological molecules with engineered materials unlocks a world of tailored applications. This strategic marriage empowers hydrogels with bioactive properties, paving the way for advancements in diagnostics, therapeutics, and regenerative medicine.

#### 5.3.1 Bioactive nanoparticle integration

Nanoparticles serve as versatile platforms for biofunctionalization. Hydrogels can be imbued with specific targeting, signaling, or recognition capabilities by conjugating biomolecules onto nanoparticle surfaces, enhancing their biocompatibility and interaction with living systems. Through the conjugation of biomolecules onto nanoparticle surfaces, hydrogels acquire the ability to interact precisely with biological systems, offering a plethora of advantages across various applications. This biofunctionalization empowers hydrogels with tailored capabilities such as targeted delivery, cell adhesion modulation, and biosensing, thus revolutionizing fields like drug delivery, tissue engineering, and diagnostics. The combination of nanoparticles' versatility and hydrogels' unique properties creates a powerful synergy that bridges the gap between synthetic materials and biological functionality, propelling advances in biomedicine and beyond.

#### 5.3.2 Tissue engineering and regeneration

Biofunctionalized hydrogels create a nurturing environment for tissue engineering and regenerative medicine. Integrating growth factors, cytokines, or extracellular matrix components into hydrogels guides cell behavior and tissue formation, facilitating wound healing and organ repair. Integrating bioactive molecules such as growth factors, cytokines, and extracellular matrix components into hydrogel matrices creates a tailored microenvironment that actively guides and influences cell behavior. This orchestrated interaction between biofunctionalized hydrogels and cells fosters tissue formation, making these hydrogels ideal scaffolds for promoting wound healing, organ repair, and tissue regeneration. The biofunctionalized hydrogel's ability to mimic the natural cellular environment holds promise for developing advanced therapies that harness the body's innate regenerative capacities, offering innovative solutions for addressing injuries, diseases, and organ deficiencies.

#### 5.3.3 Surface patterning and cell adhesion

Nanotechnology enables precise control over surface patterning within hydrogels. Hydrogel surfaces can be engineered by incorporating biofunctionalized nanoparticles to promote or inhibit cell adhesion, influencing cellular responses and creating controlled microenvironments. This remarkable capability enables the creation of controlled microenvironments where cell responses can be tailored to specific applications. By strategically engineering hydrogel surfaces to either promote or inhibit cell adhesion through the presence of biofunctionalized nanoparticles, researchers can design platforms that mimic natural cellular interactions, guide tissue development, and optimize outcomes in fields such as tissue engineering, regenerative medicine and beyond. This level of precision in controlling cell–material interactions opens new frontiers in biomedical research and therapeutic interventions.

#### 5.3.4 Targeted drug delivery

Biofunctionalization enhances the specificity of drug delivery from hydrogels. Targeting ligands immobilized on nanoparticles guide hydrogels to specific cell types or tissues, optimizing drug delivery precision and minimizing off-target effects. Hydrogels can navigate to specific cell types or tissues accurately by immobilizing targeting nanoparticle ligands. This targeted approach ensures that therapeutic agents are delivered precisely to their intended destinations, minimizing off-target effects and potential side effects. The synergy between biofunctionalized nanoparticles and hydrogels revolutionizes drug delivery by combining the controlled release capabilities of hydrogels with the selectivity and specificity conferred by the targeting ligands. This advancement can significantly improve therapeutic outcomes, reduce adverse effects, and pave the way for more efficient and patient-tailored treatments in various medical applications.

#### 5.3.5 Biosensors and diagnostics

Biofunctionalized hydrogels integrated with nanoparticles can be biosensors, detecting specific analytes or pathogens. The binding of target molecules triggers characteristic responses, enabling rapid and sensitive diagnostics for healthcare and environmental monitoring. These innovative materials can detect and respond to specific analytes or pathogens by incorporating specific biomolecules onto hydrogel nanoparticles. When target molecules bind to the biofunctionalized nanoparticles, distinct and characteristic responses are triggered within the hydrogel, allowing for rapid and sensitive detection. This unique synergy between nanoparticles and hydrogels holds immense promise for advancing diagnostics in healthcare and environmental monitoring. The resulting biosensors offer the ability to quickly and accurately identify a wide range of substances, contributing to early disease detection, real-time monitoring, and practical risk assessment in diverse applications, from medical diagnostics to environmental protection.

#### 5.3.6 Challenges and innovations

Hydrogels' successful biofunctionalization necessitates carefully considering biomolecule selection, conjugation techniques, and stability. Nanoparticle–hydrogel interactions play a crucial role in maintaining bioactivity while preserving material integrity. The successful biofunctionalization of hydrogels requires meticulous consideration of biomolecule selection, conjugation techniques, and stability. Maintaining both bioactivity and material integrity demands a profound understanding of nanoparticle–hydrogel interactions. Overcoming these challenges sparks innovations in biomolecule design, conjugation chemistries, stability enhancement, and biocompatibility testing. The quest for stability and longevity drives advancements in preserving biofunctionalized hydrogel-nanoparticle systems, ensuring their efficacy during storage and application. Furthermore, pursuing multifunctional platforms that integrate targeting, therapeutic agents, and imaging probes offers a frontier of innovation. Collaboration across disciplines, harnessing emerging technologies, and unconventional approaches are instrumental in future-proofing biofunctionalized hydrogel-nanoparticle systems, propelling transformative strides in healthcare, diagnostics, and beyond.

#### 5.3.7 Personalized medicine and beyond

Biofunctionalized hydrogels have the potential to drive personalized medicine. Tailoring hydrogels with patient-specific biomolecules creates a platform for customized therapeutic interventions, ranging from drug delivery to tissue engineering. Incorporating patient-specific biomolecules into hydrogels creates a versatile platform for tailored therapeutic interventions. This customization spans a broad spectrum, from precisely targeted drug delivery to designing tissue-engineered constructs that match individual patient needs. Biofunctionalized hydrogels serve as a dynamic interface between biomolecular specificity and material engineering, offering unprecedented opportunities to optimize treatment outcomes, minimize side effects, and enhance patient well-being. This transformative approach not only redefines how we deliver therapies but also embodies the essence of precision medicine, ushering in a new era of healthcare that revolves around the unique characteristics of each patient.

#### 5.3.8 Future directions

The exploration of biofunctionalization within the nanoparticle–hydrogel framework opens doors to pioneering applications. As advances in nanotechnology and bioconjugation techniques continue, the scope of biofunctionalized hydrogels expands, offering transformative solutions in medicine, biotechnology, and beyond. This innovative synergy creates a fertile ground for pioneering applications that transcend traditional boundaries. As nanotechnology and bioconjugation techniques advance, the horizon of biofunctionalized hydrogels broadens, promising transformative solutions across diverse domains. In medicine, these biofunctionalized hydrogels hold the potential to revolutionize diagnostics, drug delivery, tissue engineering, and regenerative therapies. The impact reverberates throughout biotechnology, enabling breakthroughs in biosensing, biomaterials, and personalized treatments. Beyond these realms, the possibilities are boundless, with potential applications reaching into environmental monitoring, energy, and beyond. The evolving landscape of biofunctionalized hydrogels embodies the limitless potential of human ingenuity, paving the way for a future where interdisciplinary collaboration and technological innovation drive profound advancements for the betterment of society.

In summary, the synergy between nanotechnology and hydrogel chemistry presents an exciting avenue for enhancing biofunctionalization. By seamlessly integrating bioactive nanoparticles into hydrogel matrices, we transcend conventional materials, forging a path toward applications that harness the intricate interplay between engineered materials and biological systems.

## 6. Biomedical applications of nanocomposite hydrogel biomaterials

Hydrogels are biocompatible, soft, and highly hydrated like native tissues, while nanoparticles impart additional functionality. Nanoparticles tuned for hydrogel compatibility enhance mechanical, electrical, magnetic, and other material aspects to suit specific tissue/organ requirements. This tailoring ability *via* nanoparticle engineering and multi-scale structure makes nanocomposite hydrogels appropriate for macro to micro-scale applications. Stimuli-responsiveness from hydrogels coupled with nanoparticle interactions enable remote triggering of drug release, cell response *etc.* Nanoparticles act as crosslinking or reinforcing hubs modulating hydrogel behavior at molecular and macroscale levels. Homogenous nanoparticle dispersion within hydrogel networks results in synergistic effects *versus* isolated components. Biomedical applications include diagnostic capabilities, drug delivery scaffolds, tissue engineering, biosensing, implant coatings, wound healing and more. Commercial translation is increasing due to optimizing properties, reproducibility, and scale-up techniques.

### 6.1. Advancing diagnostic capabilities with magnetic nanoparticle hydrogels

**Fig. 4:**
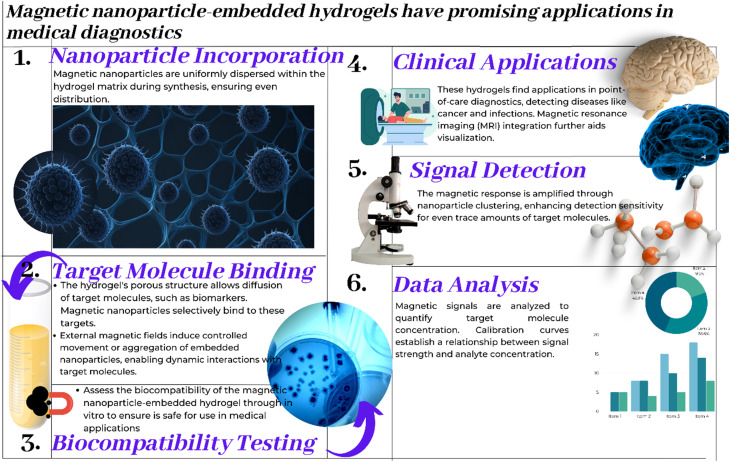
Exploring the role of magnetic nanoparticle hydrogels in diagnostics.

Integrating magnetic nanoparticles (MNPs) within hydrogel matrices has led to innovative diagnostic platforms with enhanced capabilities. This synergistic combination of MNPs and hydrogels offers unique opportunities for sensitive, specific, and versatile diagnostic applications. Fig. 4 explores the role of magnetic nanoparticle hydrogels in diagnostics. *Nanoparticle incorporation*: Fig. 4 illustrates embedding magnetic nanoparticles within a hydrogel matrix. The hydrogel structure is depicted with a network of polymer chains, and magnetic nanoparticles are distributed within the hydrogel. Arrows indicate the encapsulation process. *Target molecular binding*: Fig. 4 depicts the interaction between functionalized magnetic nanoparticle hydrogels and target molecules. The hydrogel matrix is functionalized with specific ligands or antibodies to capture the target molecules. The binding between the hydrogel-bound ligands and the target molecules is indicated with arrows. *Biocompatibility testing*: Fig. 4 shows a schematic representation of biocompatibility testing.

Cells or tissues are exposed to magnetic nanoparticle hydrogels, and their response is evaluated. Various assays and tests, such as viability assays and histological examination, are represented to demonstrate biocompatibility assessment. *Clinical application*: Fig. 4 depicts the clinical application of magnetic nanoparticle hydrogels. An imaging device (*e.g.*, MRI or MPI) is shown being used on a patient. The hydrogel-enhanced imaging of a specific target (*e.g.*, tumor) is highlighted, emphasizing the diagnostic potential in a natural clinical setting. *Signal detection*: Fig. 4 illustrates the process of signal detection using magnetic nanoparticle hydrogels. The hydrogel-bound target molecules generate a detectable signal (*e.g.*, magnetic resonance or optical signal), which a sensing device captures. The signal pathway is depicted, along with an amplification mechanism (if applicable). *Data analysis*: In Fig. 4, a representation of collected signals or images is displayed on a screen. Graphs, charts, and computational processes are included to demonstrate the extraction of meaningful information from the diagnostic signals.

**Fig. 5:**
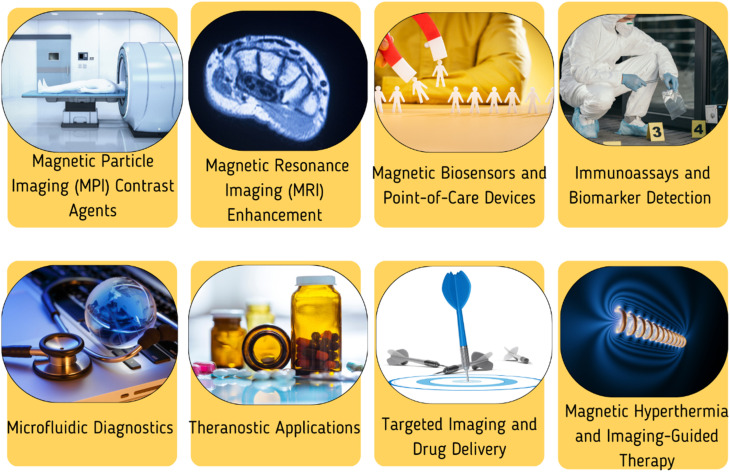
Advancing innovative diagnostic platforms for enhanced sensitivity and specificity of magnetic nanoparticle hydrogels.

The integration of magnetic nanoparticles (MNPs) within hydrogel matrices has led to the development of innovative diagnostic platforms with enhanced capabilities as illustrated in Fig. 5. This synergistic combination of MNPs and hydrogels offers unique opportunities for sensitive, specific, and versatile diagnostic applications. Here, we explore the role of magnetic nanoparticle hydrogels in diagnostics:

(1) Magnetic particle imaging (MPI) contrast agents: magnetic nanoparticle-loaded hydrogels are effective contrast agents for MPI, a novel imaging technique. These hydrogels can be designed to optimize the performance of MPI by providing a stable and concentrated source of MNPs. The hydrogel matrix helps retain the MNPs at the imaging site, improving signal quality and enhancing the accuracy of MPI for various applications, including vascular imaging and cancer detection.

(2) Magnetic resonance imaging (MRI) enhancement: hydrogels incorporating MNPs act as versatile MRI contrast agents. The hydrogel matrix provides a stable environment for the MNPs, minimizing aggregation and improving their dispersion in biological tissues. This enhances the contrast and sensitivity of MRI, allowing for more accurate anatomical and functional imaging. Additionally, the hydrogel's biocompatibility ensures minimal adverse effects.

(3) Magnetic biosensors and point-of-care devices: magnetic nanoparticle hydrogels are employed in biosensor platforms for rapid and sensitive diagnostics. Functionalized MNPs within the hydrogel selectively capture target biomolecules, triggering changes in magnetic properties that can be easily detected. These hydrogel-based biosensors find applications in point-of-care testing, allowing on-site disease detection and monitoring.

(4) Immunoassays and biomarker detection: magnetic nanoparticle hydrogels are utilized in immunoassays and biomarker detection assays. The hydrogel provides a three-dimensional network that enhances the immobilization of capture molecules (*e.g.*, antibodies), improving sensitivity and specificity. The MNPs amplify the detection signal, enabling the quantification of disease-related biomarkers.

(5) Microfluidic diagnostics: magnetic nanoparticle hydrogels are integrated into microfluidic devices, creating lab-on-a-chip platforms for diagnostics. These devices utilize the responsiveness of MNPs to external magnetic fields within the hydrogel matrix to achieve controlled sample manipulation, mixing, and analysis. This technology enables rapid and portable diagnostic testing.

(6) Theranostic applications: magnetic nanoparticle hydrogels can simultaneously serve as diagnostic agents and therapeutic carriers. Theranostic platforms allow real-time treatment response monitoring while delivering targeted therapies by incorporating diagnostic molecules and therapeutic agents within the hydrogel-MNP matrix.

(7) Targeted imaging and drug delivery: combining MNPs and hydrogels enables targeted imaging and drug delivery. MNPs functionalized with targeting ligands are embedded within the hydrogel, allowing for specific accumulation at disease sites. This approach enhances both imaging accuracy and the localized delivery of therapeutic agents.

(8) Magnetic hyperthermia and imaging-guided therapy: magnetic nanoparticle hydrogels can be employed for imaging-guided therapeutic approaches, such as magnetic hyperthermia. The hydrogel-MNP system facilitates accurate heating of tumor tissue under an alternating magnetic field, enabling localized treatment while monitoring the process through imaging. Incorporating MNPs into hydrogel matrices enhances diagnostic capabilities by improving signal sensitivity, target specificity, and spatial accuracy. However, challenges include optimizing MNP loading, ensuring proper dispersion, and achieving precise control over hydrogel properties. As research continues, magnetic nanoparticle hydrogels are promising for advancing diagnostic techniques, enabling early disease detection, and enhancing patient care.

### 6.2. Advancing tissue engineering and regenerative medicine

**Fig. 6:**
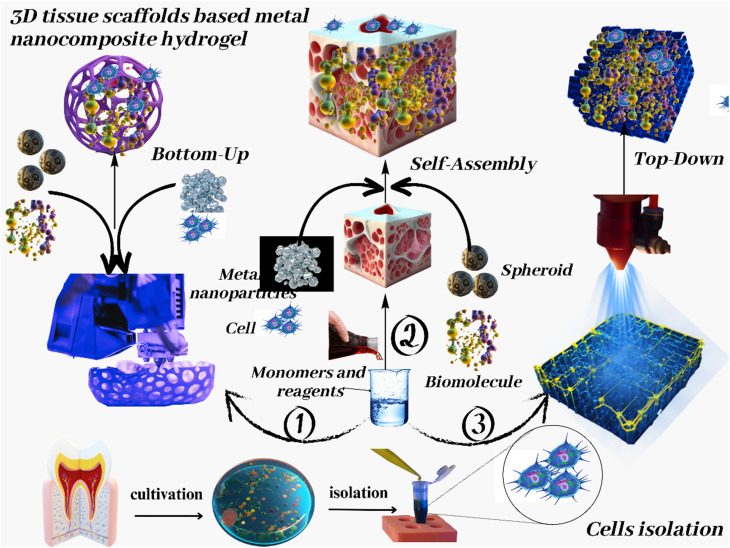
Figure proposed three routes of 3D tissue scaffolds based on meatal nanocomposite hydrogels.

Nanocomposite hydrogels show immense promise for advancing tissue engineering and regenerative medicine due to their ability to mimic the native extracellular matrix (ECM) and influence cellular behavior. Hydrogel ECM-mimetic structure and tunable mechanics/compliance provide an ideal 3D cellular microenvironment. Nanoparticles impart missing ECM functionalities like cell adhesion ligands, growth factors, or mechanical reinforcements. Functionalized nanoparticles act as bioactive cues to guide cell adhesion, proliferation and differentiation down target lineages. Degradable or stimuli-responsive nanoparticles enable the on-demand presentation/release of signals over time in 3D. Vascularized constructs become possible by incorporating nanoparticles that induce angiogenesis. Nanocomposite injectability allows minimally invasive implantation and *in situ* gelation around defects. Nanoparticles enhance the mechanical properties of load-bearing tissues and prevent scaffold collapse. Tailored properties aid interfacing with native tissues, reducing foreign body response. Nanoparticle tracking facilitates the non-invasive evaluation of construct remodeling *in vivo*. Scaffold design is crucial in tissue engineering and regenerative medicine, influencing cell adhesion, proliferation, and tissue formation. Nanocomposite hydrogels offer a versatile platform for scaffold design, combining the benefits of hydrogel matrices with the functionalities of nanoparticles. 3D tissue scaffold-based metal nanocomposite hydrogel can be assembled using three routes, as shown in Fig. 6. Incorporating metal nanoparticles adds unique properties to the hydrogel, making it suitable for various tissue engineering applications. (1) Bottom-up fabrication with metal nanocomposite hydrogels: bottom-up fabrication involves layer-by-layer assembly of the scaffold using metal nanocomposite hydrogels. Additive manufacturing methods, such as 3D bioprinting, can deposit the hydrogel layer by layer, creating a precise 3D structure. Metal nanoparticles integrated into the hydrogel can provide additional functionalities, such as electrical stimulation, which is essential for cardiac or neural tissue tissues. (2) Self-assembly of metal nanocomposite hydrogels: self-assembly utilizes the unique properties of metal nanocomposite hydrogels to form complex 3D structures spontaneously. The metal nanoparticles within the hydrogel can play a role in directing the self-assembly process through specific interactions. This approach is precious for creating intricate tissue architectures that mimic the natural extracellular matrix. (3) Top-down fabrication with metal nanocomposite hydrogels: in this approach, large structures are shaped from a bulk material that contains metal nanoparticles dispersed within the hydrogel matrix. Techniques such as laser ablation, mechanical cutting, or milling can create the desired scaffold architecture. The presence of metal nanoparticles can enhance the mechanical properties and conductivity of the scaffold, making it suitable for applications in tissues such as muscles or nerves.

Integrating metal nanoparticles within the hydrogel matrix offers advantages such as enhanced mechanical properties, electrical conductivity, and potential for localized drug delivery in each of these routes. The choice of assembly route will depend on factors such as the tissue type, the required properties of the scaffold, and the specific application within tissue engineering and regenerative medicine. Here, we delve into the specifics of scaffold design for enhancing cell adhesion and growth using nanocomposite hydrogels:

*Nanoparticle integration*: nanocomposite hydrogels are engineered by incorporating nanoparticles with specific properties into hydrogel matrices. These nanoparticles can be tailored to provide cues for cell adhesion and growth. For example, nanoparticles functionalized with cell-adhesive peptides (*e.g.*, RGD) can promote initial cell attachment and spread on the scaffold surface.

*Surface modification*: nanoparticles within the hydrogel can modify the scaffold surface to present bioactive ligands and receptor-binding sites. This modification enhances the scaffold's ability to interact with cells and stimulate adhesion. The controlled spatial distribution of nanoparticles further allows for precise control over cell–scaffold interactions.

*Growth factor delivery*: nanoparticles loaded with growth factors or signaling molecules can be dispersed within the hydrogel. This controlled release of bioactive factors promotes cell proliferation, differentiation, and tissue-specific functions. The synergistic effect of nanocomposite hydrogels and growth factor delivery enhances tissue regeneration potential.

*Mechanical properties*: nanoparticles can reinforce the mechanical properties of hydrogel scaffolds, mimicking the native tissue environment. The integration of nanoparticles can enhance the scaffold's stiffness, elasticity, and overall structural integrity, providing mechanical cues that influence cell behavior and growth.

*Electrical and conductive properties*: some nanoparticles exhibit electrical conductivity, making them valuable for scaffold design in tissues like the heart and nervous system. Nanocomposite hydrogels can provide conductive pathways facilitating cell communication and integration within electrically active tissues.

Nanoscale topography: nanocomposite hydrogels can be engineered to possess nanoscale surface topography through nanoparticle incorporation. This topography influences cell morphology, alignment, and gene expression. Cells sense and respond to these nanoscale features, affecting their adhesion and growth behavior.

*Vascularization promotion*: nanocomposite hydrogels can support vascularization by incorporating nanoparticles that release angiogenic factors or mimic the ECM of blood vessels. This facilitates the infiltration of endothelial cells and the formation of functional vascular networks within the scaffold.

*Degradation and remodeling*: nanoparticles can influence scaffold degradation and remodeling rates. By controlling the degradation kinetics through nanoparticle properties, the scaffold provides a temporary support structure while promoting new tissue formation.

*Biocompatibility and immunomodulation*: nanoparticles can be engineered to enhance scaffold biocompatibility and modulate immune responses. Surface functionalization with immunomodulatory nanoparticles minimizes inflammation and immune rejection, promoting favorable cell–scaffold interactions.

*Patient-specific tailoring*: nanocomposite hydrogels can be customized to meet patient-specific needs. Nanoparticles can carry personalized bioactive molecules, optimizing cell adhesion and growth for individual patients.

Incorporating nanoparticles within hydrogel scaffolds offers a versatile approach to tailor scaffold design for enhanced cell adhesion and growth. The synergy between hydrogel properties and nanoparticle functionalities opens exciting possibilities for creating biomimetic and bioactive scaffolds that promote tissue regeneration and drive advancements in tissue engineering and regenerative medicine.

### 6.3. Innovations in targeted drug delivery and controlled release

In recent years, the innovative capabilities of nanocomposite hydrogels have dramatically transformed the drug delivery landscape. These advancements have ushered in a new era of targeted and controlled release of therapeutic agents, introducing levels of precision and efficiency previously thought unattainable. This paradigm shift holds tremendous potential to elevate therapeutic outcomes, mitigate unwanted side effects, and ultimately redefine the boundaries of patient care. This exploration delves into the groundbreaking strategies that underpin targeted drug delivery and controlled release, all orchestrated by the dynamic interplay of nanocomposite hydrogels and nanoparticles. At the heart of this revolution lies the concept of nanoparticle-loaded hydrogel matrices. By seamlessly blending the benefits of hydrogel matrices with the unique functionalities of nanoparticles, an ingenious platform for drug encapsulation and release is born. The crux of this innovation lies in the dispersion of drug-loaded nanoparticles within the hydrogel structure, a marriage that bestows controlled and sustained release kinetics upon the therapeutic agents. This orchestrated approach to drug release preserves the stability of delicate compounds and empowers their deployment to precise anatomical locales. One of the most remarkable breakthroughs in this realm is the emergence of targeted drug delivery. Nanoparticles can be engineered to carry specific ligands on their surface within nanocomposite hydrogels. These molecular keys recognize and precisely bind to target cells, tissues, or receptors, thus orchestrating a symphony of selective drug delivery. This exquisitely targeted approach minimizes collateral damage to healthy tissue, maximizing the therapeutic impact where it is needed most while minimizing adverse effects. Harnessing the power of stimuli-responsive release, nanocomposite hydrogels adapt and respond to many external cues.

Incorporating nanoparticles finely attuned to pH, temperature, light, or enzymatic triggers results in a drug-release ballet choreographed by these precise cues. This translates into an ability to orchestrate drug release accurately, circumventing traditional limitations and ushering in a new era of on-demand drug delivery. Yet, the symphony of innovation does not end here. Nanocomposite hydrogels present an opportunity for dual-drug delivery, an ingenious strategy wherein nanoparticles harboring distinct therapeutic agents can be embedded within the hydrogel scaffold. This ingenious symphony of co-release has the potential to synergistically address complex medical conditions that may require a multifaceted approach.

Further expanding the realms of possibility is the concept of sequential release. By integrating nanoparticles with varying properties, nanocomposite hydrogels can serve as a stage for releasing drugs in a carefully orchestrated sequence. This sequential release strategy can potentially optimize therapeutic regimens and elegantly address the dynamic nature of disease processes. Their role as long-acting depot formulations further exemplifies the versatility of nanocomposite hydrogels. In the case of drugs with short half-lives, these hydrogels stand as stalwart sentinels, releasing therapeutic agents in a sustained and controlled manner over an extended period. This is not only a testament to the ingenuity of the technology but also a nod to the improved patient compliance that ensues. Intriguingly, nanocomposite hydrogels can also venture into intracellular delivery.^101^ Functionalized nanoparticles within these hydrogels can facilitate the penetration of cellular barriers, ushering in a new era of intracellular drug delivery that holds tremendous potential for treating diseases that have thus far evaded therapeutic intervention.

As the field of medicine continues to stride toward a future defined by precision, the advent of personalized medicine becomes increasingly tantalizing. Nanocomposite hydrogels, with their ability to incorporate patient-specific nanoparticles and drugs, are ideally poised to carry the torch of personalized drug delivery. This harmonious union of technology and individualized care can usher in an era where treatment strategies are as unique as the patients they serve. Looking to the future, the capabilities of nanocomposite hydrogels promise to transcend the boundaries of traditional drug delivery paradigms. A particularly intriguing innovation lies in the realm of remote-controlled release. By leveraging external stimuli such as magnetic fields or ultrasound, these hydrogels offer a non-invasive means of triggering drug release at targeted sites. This level of spatiotemporal control over drug delivery has profound implications for treatment precision and efficacy. Nanocomposite hydrogels also bear the potential for theranostic applications in a breathtaking fusion of diagnostic and therapeutic prowess. Here, nanoparticles within the hydrogel scaffold are entrusted with a dual role: they ferry therapeutic agents and serve as emissaries of real-time imaging. The symbiotic relationship between treatment and monitoring introduces an unprecedented refinement to patient care.

In conclusion, the confluence of nanocomposite hydrogels and nanoparticle science has ushered in a new era of drug delivery and controlled release. The orchestrated ballet of targeted delivery, stimuli-responsive release, and personalized medicine holds the promise of transforming medical treatments across the spectrum. These innovations are poised to revolutionize therapeutic strategies, propelling us into an era where precision, efficacy, and patient well-being are paramount. As we journey forward, the horizon is ablaze with the potential of nanocomposite hydrogels, forever altering how we deliver health and healing.

### 6.4. Responsive hydrogels for on-demand drug release

**Fig. 7:**
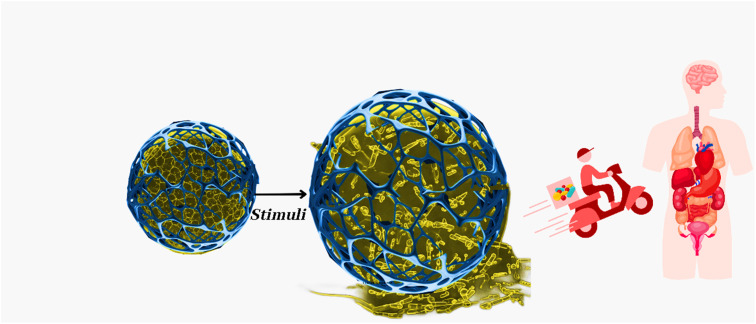
Figure shows the external environment's effect on the drug released from hydrogel.

Responsive hydrogels are at the forefront of innovative drug delivery systems, enabling on-demand drug release in response to specific stimuli. When combined with nanoparticles, these smart hydrogels offer a sophisticated platform for precise and controlled therapeutic delivery. Here, we delve into responsive hydrogels for on-demand drug release, focusing on their design and applications: Fig. 7 demonstrates the hydrogel volume change after exposure to external stimulus.

Nanocomposite hydrogels hold immense potential as a platform for localized delivery of cancer therapeutics. By embedding drug-loaded nanoparticles within a responsive hydrogel matrix, these innovative delivery systems provide targeted accumulation of drugs at tumor sites while enabling sustained release profiles to maximize treatment duration. Their versatility allows for loading single or combination therapies to overcome resistance. When functionalized with tumor-targeting ligands, nanoparticles preferentially accumulate in cancers due to the enhanced permeability and retention effect, concentrating payloads where needed. One significant advantage of this approach is minimizing systemic exposure to toxic chemotherapy agents. By localizing drug release to tumors, nanocomposite hydrogels help spare healthy tissues from damage and reduce adverse side effects that compromise patients' quality of life. This enhances the tolerability of intense cancer regimens. Minimally invasive administration routes like injections further boost patient comfort levels. A key attribute is the capacity for sustained release at the disease site. Nanoparticles encapsulated within the hydrogel matrix gradually release drugs over time to maintain therapeutic concentrations. This overcomes the limitations of traditional bolus injections that fail to achieve steady drug levels. Prolonged, controlled dosing is critical, given the need for long-term cancer management.

Combination nanoparticle payload designs allow simultaneous or sequential release of multi-drug regimens tailored for individual tumor profiles. Together with the choice of targeting moieties, hydrogels enable personalized localized therapies. Loading various agents attacking complimentary pathways boosts chances of overcoming resistance. Their pliability also accommodates immunomodulators to strengthen immune responses against cancer cell evasion mechanisms. An emerging application utilizes light-triggered designs for non-invasive remote activation of the release. Near-infrared illumination of light-sensitive nanoparticles embedded in hydrogels deposited near tumors provides a minimally invasive activation method compared to repeat surgical interventions. This offers flexibility in tailoring release timing. Imaging agent-loaded nanoparticles in the matrix also permit real-time monitoring of distribution, dosage consistency and response to combination regimens.

Additionally, bioresponsive variants activated by tumor microenvironment cues like acidic pH or enzymes promote site-specific release. pH-responsive nanoparticles maintained release below the tumor threshold but accelerated dramatically in acidic tissue, demonstrating potential. Future engineering may create even more sophisticated disease-microenvironment-activated systems. Stimuli-independent forms provide sustained constitution levels for a durable treatment effect. Ongoing research optimizes particle formulation, hydrogel porous architecture and ligand choices. Preclinical studies demonstrate significant tumor growth inhibition and survival benefits *versus* single drugs. The early clinical translation shows biocompatibility and localization within tumors. Scale-up challenges include maintaining effects at production scales while navigating regulatory clearance pathways. Long-term stability assessment and comprehensive safety/toxicology profiles must be addressed before widespread adoption. As the field matures, localized nanocomposite hydrogel delivery is primed to make significant headway in oncology. Combining tumor targeting, tunable release profiles and potential combination payloads in a minimally invasive package provides an ideal platform for personalized localized therapies. Their flexible framework accommodates developments in drug classes, delivery technologies and findings from other treatment arenas. With continued advances, this strategy promises to transform cancer management by maximizing the benefit of existing drugs while overcoming resistance challenges more effectively than ever before.

### 6.5. Dual-drug loading strategies for synergistic effects

The realm of drug delivery has been invigorated by the emergence of dual-drug loading strategies within nanocomposite hydrogels, a transformative approach that taps into synergistic effects to achieve enhanced therapeutic outcomes. This innovative paradigm entails the incorporation of two distinct therapeutic agents within a single hydrogel matrix, offering the ability to release drugs simultaneously or sequentially, thereby capitalizing on their complementary or synergistic mechanisms of action. This exploration delves deeply into the intricate world of dual-drug loading strategies within nanocomposite hydrogels, unraveling their multifaceted potential and far-reaching applications. At the heart of this innovation lies the concept of simultaneous drug release. Nanocomposite hydrogels are ingeniously designed to encapsulate two drugs, each within its dedicated set of nanoparticles. These hydrogels embark on a dual-drug delivery voyage upon administration, releasing both therapeutic agents in harmonious unison. The beauty of this approach lies in its orchestration of a synergistic interplay, where the combined efforts of the two drugs target multiple pathways or molecular targets. This orchestration is invaluable when confronting complex diseases or drug-resistant conditions, as it provides a multi-pronged assault on the ailment. Taking a step further, the strategy of sequential drug release offers a nuanced dance within the realm of dual-drug loading. Here, nanoparticles carrying one of the therapeutic agents are meticulously embedded within the hydrogel matrix, while the second agent is directly integrated. This strategic arrangement gives rise to a carefully choreographed sequence of drug release, where the initial drug sets the stage as a primer or sensitizer. The subsequent release of the second drug then capitalizes on this preparatory phase, amplifying its efficacy. This sequential ballet of drug release enhances therapeutic impact and opens doors to tailoring treatment regimens with precision. The potency of dual-drug loading strategies stems from the convergence of complementary mechanisms of action. Nanocomposite hydrogels can be ingeniously designed to facilitate a harmonious synergy between the two encapsulated drugs. Picture a scenario where one drug diligently inhibits a specific signaling pathway while its partner augments apoptosis or primes an immune response. The symphony of their combined efforts orchestrates a therapeutic crescendo that is both more potent and exquisitely targeted. A particularly intriguing aspect of dual-drug loading is its potential to combat drug resistance head-on. This innovative approach can thwart the resilience of resistant cell populations by simultaneously engaging multiple pathways or circumventing resistance mechanisms. By intertwining drugs with distinct modes of action, the nanocomposite hydrogel delivers a strategic blow to drug-resistant cells, reinvigorating treatment effectiveness and extending the armamentarium against challenging diseases. In the era of personalized medicine, dual-drug loading within nanocomposite hydrogels emerges as a beacon of hope. This approach facilitates tailoring treatment strategies to patients' unique disease profiles and sensitivity. By selecting drugs that align with an individual's specific needs, the hydrogel becomes a personalized therapeutic agent designed to optimize efficacy and outcomes for every patient. The marriage of dual-drug loading and nanocomposite hydrogels yields another remarkable dividend: the potential for minimized side effects. By harnessing the synergistic effects of drug combinations, it becomes possible to administer lower individual drug doses, thus reducing the specter of side effects and toxicity often associated with higher dosages. This newfound equilibrium between therapeutic effectiveness and patient tolerability contributes to improved compliance and patient well-being.

The allure of dual-drug loading strategies extends readily to complex diseases, with cancer as a prominent exemplar. In cancers where intricate cellular pathways conspire to drive disease progression, the ability to target these pathways simultaneously or sequentially is nothing short of revolutionary. Nanocomposite hydrogels wielding dual-drug loading strategies thus emerge as potent allies in the battle against complex diseases, promising unprecedented therapeutic impact. The canvas of dual-drug loading within nanocomposite hydrogels is expansive and capable of accommodating both dual-agent and versatile combination therapies. These hydrogels evolve into a dynamic platform for multifaceted treatment approaches by introducing multiple nanoparticles harboring diverse drug payloads. This adaptability empowers clinicians and researchers to explore and fine-tune an array of therapeutic possibilities, further widening the scope of therapeutic interventions.

Beyond its treatment potential, the dual-drug loading paradigm enables an intriguing avenue for monitoring and control. Precise tuning and monitoring of release kinetics grant the power to navigate the therapeutic journey precisely, optimizing drug ratios and release profiles to unlock optimal synergistic effects. This fine-tuning capability ensures therapeutic interventions align with patient needs and disease dynamics.

In the grand tapestry of medical advancement, dual-drug loading strategies within nanocomposite hydrogels emerge as a beacon of hope, illuminating the path toward enhanced therapeutic interventions. By melding the powers of two distinct drugs and harnessing the capabilities of nanoparticles and hydrogel matrices, this innovative approach offers a versatile solution for tackling challenging diseases, reshaping treatment paradigms, and advancing the frontiers of personalized medicine. As research and innovation march forward, dual-drug loading stands as a testament to the relentless pursuit of optimal patient outcomes and the ceaseless evolution of medical science. Tiwari *et al.*^102^ investigate the potential of using functionalized graphene oxide as a nanocarrier for dual drug delivery of quercetin and gefitinib to treat ovarian cancer. Graphene oxide has excellent properties, making it a promising candidate for drug delivery applications.

The researchers modified graphene oxide by grafting it with polyvinylpyrrolidone to improve its solubility and biocompatibility. They successfully loaded both quercetin and gefitinib onto the polyvinylpyrrolidone-functionalized graphene oxide nanocarrier. The dual-drug system showed a higher loading capacity and release rate than the single-drug systems. The researchers then tested the cytotoxicity of the drug-loaded nanocarriers on ovarian cancer cells and normal ovarian cells. They found that the dual drug-loaded nanocarrier showed significantly higher toxicity towards the ovarian cancer cells than the single drug-loaded nanocarriers and the free drugs. However, it did not show significant cytotoxicity towards the normal ovarian cells.

In summary, the polyvinylpyrrolidone-functionalized graphene oxide nanocarrier could efficiently load and release the dual drug system of quercetin and gefitinib. The dual drug-loaded nanocarrier showed enhanced anticancer activity against ovarian cancer cells with low cytotoxicity toward normal cells. So, this dual-drug delivery system holds promise for improved ovarian cancer therapy. W. Tan, *et al.*^103^ have focused on developing a groundbreaking approach to address the intricate challenges of diabetic wound healing. Through their innovative work, they have created a dual-drug loaded polysaccharide-based self-healing hydrogel, coined OCM@P hydrogel, that holds immense promise in revolutionizing the treatment of diabetic wounds.

The foundation of the OCM@P hydrogel lies in the meticulous blending of carboxymethyl chitosan and oxidized hyaluronic acid, facilitated by dynamic imine bonds and electrostatic interactions. This harmonious amalgamation is the bedrock for integrating two potent therapeutic agents, metformin and curcumin. These agents are seamlessly incorporated into the hydrogel matrix, giving rise to a multifunctional platform poised to tackle the multifaceted aspects of diabetic wound healing.

The inherent structure of the OCM@P hydrogel boasts a remarkable homogeneity and porosity, complemented by robust mechanical properties and the ability to self-heal. This combination of attributes ensures efficient wound coverage and underscores the hydrogel's potential as a robust wound dressing. The hydrogel exhibits low cytotoxicity, rapid hemostasis, favorable blood compatibility, and a formidable antibacterial prowess.

The phenomenon of “synergistic effect” is palpable within the OCM@P hydrogel. Metformin's release, characterized by an initial burst followed by a sustained pattern, synergizes with curcumin's prolonged and controlled release. This orchestrated release profile is a testament to the harmonious interplay of these therapeutic agents within the hydrogel matrix.

Moreover, the OCM@P hydrogel showcases its prowess in combating the intricacies of diabetic wound healing. It manifests exceptional extracellular and intracellular antioxidant activity, exhibiting potent anti-inflammatory effects. This duality of action contributes to mitigating oxidative stress and inflammation, paving the way for pivotal wound healing processes such as re-epithelialization, granulation tissue formation, collagen deposition, and angiogenesis.

The OCM@P hydrogel represents a paradigm shift in wound healing, particularly diabetes. By harnessing the synergistic effects of metformin and curcumin, coupled with the hydrogel's self-healing nature, a comprehensive approach to diabetic wound treatment emerges. This innovative solution holds the potential to transcend the current landscape and redefine diabetic wound care, ultimately leading to improved patient outcomes. In summation, the work by Tan and colleagues^103^ epitomizes the notion of “synergistic effect” through the marriage of therapeutic agents within a self-healing hydrogel matrix. This synergistic potency has the transformative capacity to accelerate diabetic wound healing and positions the OCM@P hydrogel as a promising candidate for effective wound dressing in treating chronic diabetic wounds.

Incorporating biopolymer nanoparticles into hydrogels can offer several potential benefits. Here are some of the benefits mentioned in the search results: improved properties: the incorporation of biopolymer nanoparticles can enhance the (bio)chemical, thermal, mechanical, and electrical properties of hydrogels. This improvement is attributed to better interactions between the nanoparticles and the polymeric chains, resulting in superior performance of the nanocomposite hydrogels. Enhanced biomedical applications: biopolymer nanoparticle–hydrogel composites have shown promise in various biomedical applications. They can enable targeted drug delivery, reduce toxicity, enhance drug solubility, and offer tunable properties. These advancements can lead to improved therapeutic outcomes and reduced side effects. Superior physical and chemical properties: compared to conventional polymer hydrogels, biopolymer nanoparticle–hydrogel composites exhibit superior physical, chemical, mechanical, and electrical properties. This can expand their potential applications in tissue engineering, regenerative medicine, and biosensing. Tunable properties: incorporating biopolymer nanoparticles allows for tuning specific properties of hydrogels, such as mechanical strength and responsiveness. This tunability enables customization based on the desired application requirements. Increased elastic moduli: incorporating nanoparticles into hydrogels can lead to enhancements in the elastic moduli of the hydrogels. This improvement in mechanical properties can benefit applications requiring higher stiffness or load-bearing capabilities.

In conclusion, incorporating biopolymer nanoparticles into hydrogels can enhance their properties, expand their applications, and improve their performance in various biomedical and materials science fields. Based on the previous literature, no specific biopolymer nanoparticles that are particularly effective in hydrogels are mentioned in the articles. However, the articles discuss using various biopolymer-based hydrogels, such as natural proteins (*i.e.*, fibrin, silk fibroin, collagen, keratin, and gelatin).^104^

### 6.6. Nanocomposite hydrogels in wound healing and tissue repair

Nanocomposite hydrogels have emerged as a remarkable class of biomaterials, holding immense promise in wound healing and tissue repair. These innovative materials seamlessly combine nanoparticles' distinctive attributes with hydrogels' therapeutic prowess, creating a versatile platform poised to revolutionize regenerative medicine. In this comprehensive exploration, we embark on a journey to unveil the multifaceted applications and profound benefits that nanocomposite hydrogels bring to the forefront of wound healing and tissue repair strategies. At the epicenter of this innovative paradigm lies the concept of accelerated healing, a pivotal objective in medical care. Nanocomposite hydrogels are designed to act as orchestrated healing agents by facilitating the controlled release of growth factors, cytokines, and other bioactive molecules. These agents, safely harbored within the matrix of nanoparticles, are gradually released into the wound environment. This orchestrated release sets the stage for a symphony of cellular responses, including enhanced cell migration, proliferation, and tissue regeneration. The harmonious interplay of these therapeutic agents within the hydrogel matrix orchestrates an accelerated healing process that holds the potential to redefine treatment outcomes. Incorporating antimicrobial nanoparticles represents a powerful innovation within the intricate tapestry of wound care. The strategic integration of these nanoparticles imparts a heightened level of antibacterial properties to nanocomposite hydrogels. This translates into a robust defense mechanism against potential infections at the wound site. By creating an environment inherently hostile to bacterial colonization, these hydrogels contribute significantly to minimizing the risk of complications, thus ensuring a sterile milieu inherently conducive to healing. Beyond their antimicrobial capabilities, nanocomposite hydrogels are adept at assuming the role of a scaffold and support system for regenerating tissues. Functioning as three-dimensional scaffolds, these hydrogels offer mechanical support to the wound area, emulating the natural extracellular matrix. This mimetic structure provides a favorable milieu for cells to adhere, proliferate, and migrate, thereby setting the stage for the highest caliber tissue regeneration. In essence, these hydrogels lay the foundation for nature to weave its intricate tapestry of healing. The ingenious incorporation of nanoparticles introduces an element of controlled drug delivery, which lies at the heart of modern therapeutics. Nanocomposite hydrogels facilitate targeted and sustained drug release with their finely tuned responsiveness to pH, enzymes, or temperature cues. This finely orchestrated release mechanism ensures therapeutic agents are delivered precisely where needed, optimizing wound healing. It is through this strategic interplay between nanoparticles and hydrogel matrices that the stage is set for a harmonious and optimized healing journey. An indispensable element of the wound healing narrative is angiogenesis, forming new blood vessels. Nanocomposite hydrogels, with their exquisite composition, can stimulate angiogenesis within the wound area. This critical process is the lifeline for healing tissues, supplying the much-needed oxygen and nutrients to facilitate accelerated regeneration. By orchestrating the dance of angiogenesis, these hydrogels contribute significantly to expediting the healing journey.

Nanocomposite hydrogels, armed with their distinctive nanoparticle constituents, are uniquely poised to modulate the inflammatory response—an essential facet of the healing process. By finely tuning the hydrogel's composition, these innovative biomaterials can tip the scales toward a balanced immune reaction that fosters healing while curbing excessive inflammation. This artful modulation of the inflammatory response emerges as a powerful strategy for promoting efficient tissue regeneration. Another remarkable facet of nanocomposite hydrogels is their potential to influence collagen deposition and remodeling. Collagen, a key player in wound healing, can be subtly guided by the controlled release of bioactive agents from the hydrogel matrix. This orchestrated dance may hold the key to reducing scar formation, offering a pathway to more organized tissue regeneration and improved cosmetic outcomes. Chronic wounds, particularly those afflicting diabetic patients, present a formidable challenge in wound care. Nanocomposite hydrogels, with their multifunctional capabilities, offer a beacon of hope in addressing these challenging scenarios. By harnessing their unique attributes, these hydrogels navigate the complexities of impaired wound healing in diabetic individuals, offering a pathway to improved outcomes and enhanced quality of life. In the landscape of personalized medicine, nanocomposite hydrogels unveil their potential to shine as bespoke healing agents. Tailored to accommodate patient-specific needs, these hydrogels can be crafted to suit wound types, sizes, and locations, ushering in a new era of treatment precision. This personalized approach ensures that the therapeutic journey is optimized to align with the unique requirements of each patient. Advancements in wound healing are not confined to the realm of therapeutics alone. Nanocomposite hydrogels, in their ingenuity, can be harnessed to provide real-time monitoring of wound healing progress. By incorporating intelligent nanoparticles into their matrix, these hydrogels offer a conduit for healthcare providers to track and assess the healing journey remotely. This real-time feedback loop empowers medical professionals to adjust treatment strategies following evolving patient needs.

The convergence of nanoparticles and hydrogel matrices within nanocomposite hydrogels unveils a treasure trove of potential to revolutionize wound healing and tissue repair strategies. These advanced biomaterials are poised to become a cornerstone of regenerative medicine, offering a multifaceted toolkit for orchestrating efficient wound closure, fostering tissue regeneration, and, ultimately, ushering in a new era of improved patient outcomes. Ghobashy *et al.*^105^ investigated a pH-sensitive hydrogel based on 2-(dimethylamino)ethyl methacrylate and polyethylene oxide crosslinked using gamma irradiation. The aim was to develop an intelligent wound dressing material that can release drugs in a pH-controlled manner. First, the hydrogel was synthesized at different irradiation doses to study its properties.

The gel fraction and swelling degree increased with higher doses due to more crosslinking. They found that the hydrogel exhibited pH-sensitive swelling behavior, absorbing more water at pH 7 than at pH 4. Next, they incorporated zinc sulfide (ZnS) nanoparticles into the hydrogel to form a nanocomposite. They loaded this nanocomposite hydrogel with three antibiotic drugs: colistin, gentamicin, and neomycin. They found the drug release was pH-dependent, with colistin and neomycin releasing more at pH 4 and gentamicin releasing more at pH 7. The nanocomposite hydrogel also showed good antimicrobial activity against bacteria and fungi, with the zinc sulfide nanoparticles enhancing the activity of the antibiotic drugs. The hydrogel loaded with neomycin showed the highest antimicrobial effect.

**Fig. 8:**
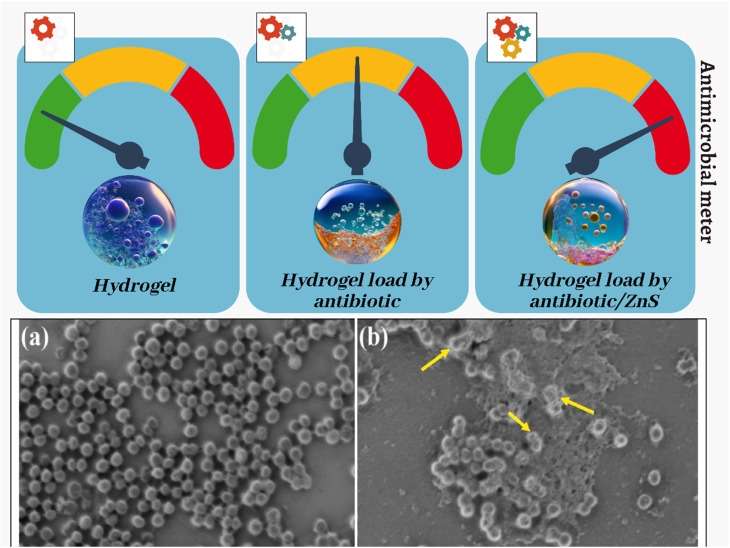
Figure illustrates the investigation into the reaction mechanism of the (DMAEM/PEO)/ZnS hydrogel loaded with neomycin, employing Scanning Electron Microscopy (SEM) analysis against the pathogenic bacterium *S. aureus*. (a) The SEM micrograph portrays the untreated control *S. aureus* cells. The bacterial groups exhibit a characteristic arrangement, adhering uniformly across the regular surface. This depiction provides a baseline reference for the bacterial cell morphology and surface interaction. (b) Presents the SEM analysis of *S. aureus* cells following treatment with the (DMAEM/PEO)/ZnS hydrogel loaded with neomycin. The treated bacterial cells exhibit striking and unusual morphological irregularities compared to the untreated control cells. The most prominent observation is the semi-lysis of the outer surface of specific bacterial cells, characterized by evident deformations and structural changes.

The pH-sensitive hydrogel nanocomposite provided controlled drug release in response to pH changes. The authors believe this makes it promising as a wound dressing material that can release drugs when needed to promote wound healing. Scanning Electron Microscopy (SEM) analysis was employed to elucidate the potential antimicrobial mechanism of action exerted by the (DMAEM/PEO)/ZnS hydrogel against the pathogenic bacterium *S. aureus*. In this investigation, SEM was utilized to visualize the interactions between bacterial cells and the hydrogel, shedding light on the antimicrobial effects. The SEM examination of control bacterial cells revealed a typical arrangement wherein bacterial groups adhered uniformly to the entire regular surface, as depicted in Fig. 8a.

Upon subjecting the (DMAEM/PEO)/ZnS hydrogel loaded with neomycin, intriguing and aberrant morphological transformations were evident in *S. aureus*, as evidenced in Fig. 8b. These transformations included the semi-lysis of the outer surface in select bacterial cells, characterized by notable deformations in the structure of the *S. aureus* cells. This observation underscores the profound impact of the (DMAEM/PEO)/ZnS hydrogel treatment on the structural integrity of the bacterial cells. In the case of the ZnS NPs neomycin hydrogel, a particularly remarkable outcome emerged. The hydrogel induced complete lysis of the bacterial cells, leading to a malformation of the cellular structure. This profound alteration in cellular morphology was accompanied by a reduction in the total viable bacterial count, as depicted in Fig. 8b.

Furthermore, the SEM images vividly depicted the creation of distinctive perforations on the surface of the bacterial cells. These findings corroborated the membrane leakage assay and visually represented the hydrogel-induced disruption of bacterial cell membranes. The SEM analysis thus served as a powerful tool to unravel the antimicrobial mechanism of the (DMAEM/PEO)/ZnS hydrogel. The observed irregularities in bacterial cell morphology, ranging from semi-lysis to complete lysis, underscored the potent antimicrobial activity of the hydrogel. The formation of holes on the bacterial cell surface further substantiated the membrane disruption effect of the hydrogel, consistent with the results of the membrane leakage assay. Overall, SEM analysis provided compelling visual evidence of the (DMAEM/PEO)/ZnS hydrogel's ability to induce structural alterations and membrane damage in *S. aureus* cells, shedding light on its antimicrobial mode of action.

## 7. Antibacterial properties of nanocomposite hydrogel for infection prevention

Nanocomposite hydrogels have emerged as a promising and innovative solution to address a critical aspect of wound care: infection prevention. Leveraging the inherent antibacterial properties of nanoparticles, these advanced biomaterials have paved the way for a multifaceted approach to combating bacterial infections and facilitating effective wound healing. In this context, we delve into utilizing nanocomposite hydrogels with antibacterial properties as a proactive strategy for infection prevention in wound care. At the heart of this approach is incorporating antimicrobial nanoparticles, such as silver, zinc oxide, or copper nanoparticles, within the nanocomposite hydrogel matrix. These nanoparticles serve as reservoirs for antimicrobial agents, which are gradually released to create a hostile environment for a broad spectrum of bacteria. These hydrogels act as a formidable barrier against infection by thwarting bacterial growth and colonization. One of the notable advantages of this approach is the ability of antibacterial nanoparticles to disrupt bacterial biofilms. These complex structures, consisting of bacteria encased in a protective matrix, pose a significant challenge due to their resistance to traditional antibiotics. The nanocomposite hydrogels effectively dismantle these biofilms, mitigating the risk of chronic infections and promoting a conducive environment for wound healing. Notably, the integration of antibacterial nanoparticles within nanocomposite hydrogel dressings translates into a reduced risk of infection in various wound scenarios. Whether it's a surgical incision, burn, or chronic ulcer, these hydrogels offer an added layer of protection that can stave off complications and expedite the healing process. A key attribute that sets nanocomposite hydrogels apart is their ability to sustain the release of antimicrobial agents over an extended period. This sustained release ensures a continuous and consistent protective effect at the wound site, preventing bacterial resurgence and maintaining an environment conducive to healing.

Moreover, the multifaceted mechanism of action exhibited by antibacterial nanoparticles reduces the likelihood of bacterial resistance development, a concern that looms large in the era of increasing antibiotic resistance. What makes nanocomposite hydrogels even more intriguing is their potential for personalized treatment. By tailoring the composition and concentration of antimicrobial nanoparticles to match the bacterial profile of each wound, these hydrogels offer targeted and effective infection prevention, enhancing the precision of wound care strategies. Beyond their antibacterial prowess, nanocomposite hydrogel dressings offer many additional benefits. Their ability to retain moisture, reduce scarring, and adhere effectively to wounds contributes to a holistic approach to wound healing. These dressings are particularly valuable in managing chronic wounds, where infection prevention is paramount. Combining antibacterial properties with other therapeutic functionalities, such as promoting angiogenesis, cell proliferation, and anti-inflammatory effects, nanocomposite hydrogel dressings present a multifunctional approach to wound healing support. This comprehensive strategy addresses various facets of wound healing, further bolstering its potential impact.

**Fig. 9:**
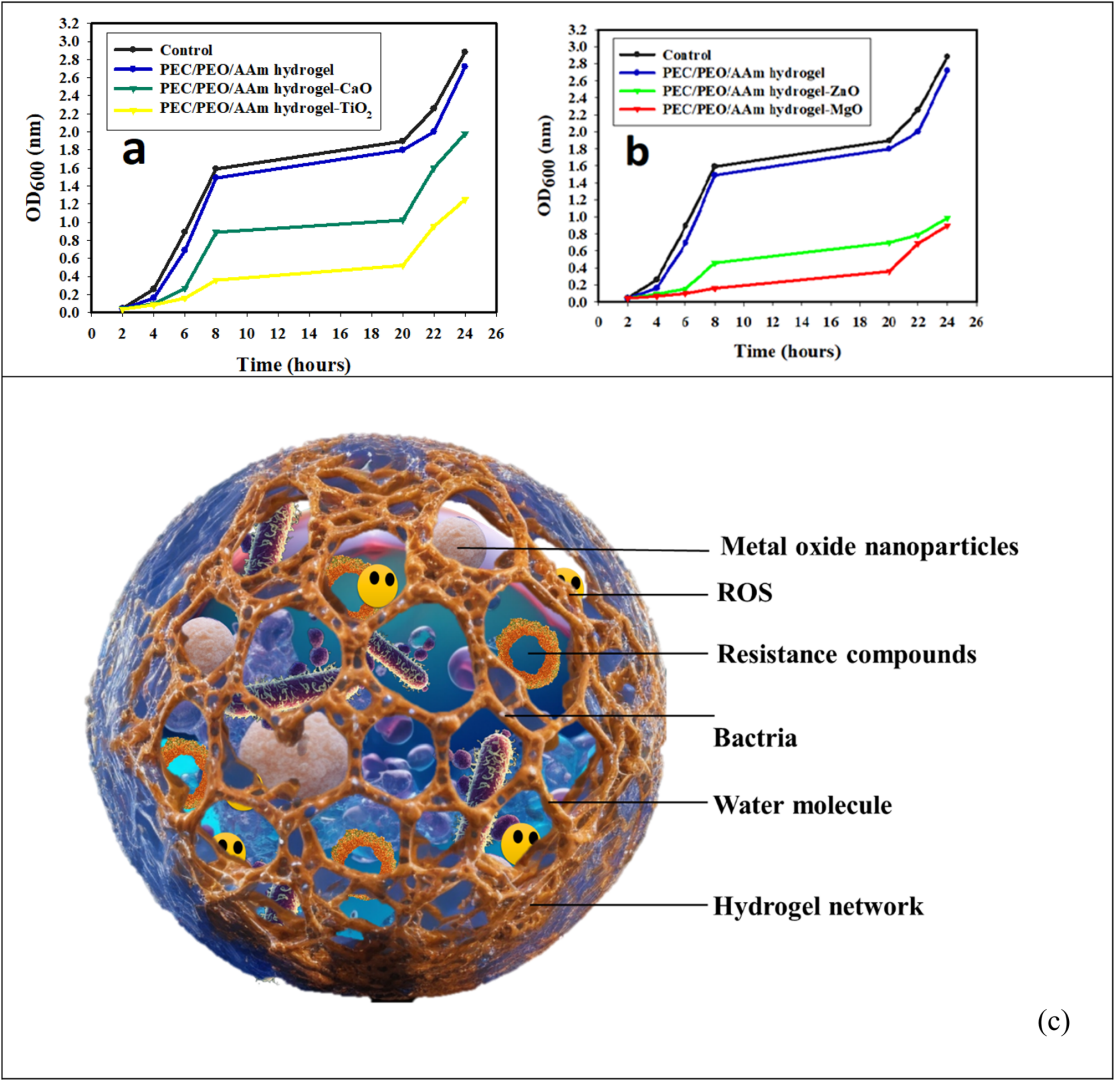
Impact of PEC/PEO/AAm hydrogel, modified with CaO and TiO_2_ (a), and PEC/PEO/AAm hydrogel, modified with ZnO and MgO (b), on *S. aureus* Growth curve and (c) synergistic antibacterial mechanism of nanoparticles.

From a clinical perspective, adopting nanocomposite hydrogel dressings with antibacterial properties can yield profound benefits. Healthcare-associated infections can be significantly reduced, leading to shorter hospitalization durations, lower treatment costs, and improved patient outcomes. Integrating antibacterial properties within nanocomposite hydrogel dressings is a monumental advancement in wound care, underscoring the potential to reshape the landscape of infection prevention and wound healing. Integrating bioactive nanoparticles within nanocomposite hydrogels holds transformative potential in wound healing and tissue repair, particularly in growth factor delivery. This innovative approach combines the regenerative power of growth factors with the controlled release attributes of nanoparticles and hydrogel matrices, culminating in a platform that expedites the healing process and fosters tissue regeneration. In this exploration, we delve into bioactive nanoparticles as conduits for growth factor delivery within the intricate landscape of nanocomposite hydrogels. Central to this strategy is the encapsulation of growth factor-loaded nanoparticles within the nanocomposite hydrogel matrix. These nanoparticles function as guardians, safeguarding the integrity of growth factors against enzymatic degradation and orchestrating a gradual and sustained release. This orchestrated release ensures a consistent and uninterrupted supply of healing cues to the wound site, setting the stage for an accelerated healing trajectory. A distinct advantage of this approach lies in the heightened stability and bioavailability of growth factors conferred by nanoparticle encapsulation. By shielding against enzymatic threats, the nanocomposite hydrogel system maintains the bioactivity of growth factors, magnifying their therapeutic efficacy and impact. Intriguingly, nanocomposite hydrogels provide a canvas for the meticulous choreography of growth factor release. The controlled and sustained liberation of growth factors, orchestrated by nanoparticles, fosters an environment where cell migration, proliferation, and tissue regeneration thrive harmoniously. Abou El Fadl *et al.*^106^ investigate the antibacterial properties of hydrogels containing different metal oxide nanoparticles. The team prepared a pectin/polyethylene oxide-based hydrogel using gamma irradiation. They then incorporated titanium dioxide, magnesium oxide, calcium oxide and zinc oxide nanoparticles into the hydrogel matrix. The nanocomposites were characterized using techniques like FTIR, XRD, and TEM. The FTIR analysis showed that incorporating metal oxides caused changes in the spectrum, indicating interactions between the nanoparticles and the hydrogel. The XRD patterns confirmed the presence of specific crystallographic planes corresponding to the metal oxides, showing they were successfully incorporated. TEM and SEM images revealed details about the morphology, size, and distribution of the nanoparticles within the hydrogel. The antibacterial activity of the hydrogel nanocomposites was evaluated against bacteria and fungi. The results showed that the magnesium oxide and zinc oxide hydrogels exhibited significant inhibitory effects, suggesting potential as antimicrobial agents. The hydrogel containing magnesium oxide nanoparticles showed the greatest antibacterial activity against *Staphylococcus aureus*, as seen in Fig. 9a and b growth curve tests. The synergistic effects between the hydrogel matrix and metal oxide nanoparticles likely contributed to the enhanced antibacterial activity. The hydrogel network helps maximize the nanoparticles' effects by increasing contact with bacterial cells, absorbing resistance compounds, and supplying water for ROS generation. Incorporating metal oxide nanoparticles, especially magnesium oxide, significantly enhanced the hydrogel's antimicrobial properties. A four-step synergistic pathway unveiled in Fig. 9c for enhanced antimicrobial activity: (1) the hydrogel matrix serves as a platform for metal oxide nanoparticles to adhere to bacterial cells, significantly increasing the contact and interaction between the nanoparticles and bacteria. The three-dimensional network of the hydrogel physically entraps bacteria, bringing them close to the nanoparticles. This proximity facilitates robust interactions and enables the effective delivery of antimicrobial effects. (2) Metal oxide nanoparticles attached to bacterial surfaces penetrate the cells through two distinct mechanisms: membrane permeation or electrostatic interactions. This internalization results in a temporary and localized surge of nanoparticle concentration both on the cell surface and within the bacterium. (3) The hydrogel matrix possesses the unique ability to absorb or sequester resistance compounds released by bacteria. This action diminishes the resistance effect, amplifying the antimicrobial activity of the nanocomposites. The hydrogel's porous structure and high water content provide an ideal environment for absorbing and entrapping molecules, including the resistance compounds. This process helps curtail the availability of resistance agents and counteracts their influence on the nanoparticles. (4) Nanoparticles, notably metal oxides like ZnO, MgO, CaO, and TiO_2_, operate as potent agents causing bacterium toxicity. They interact with proteins and DNA molecules within the bacterial cell walls and interiors, leading to functional disruption and eventual bacterial demise. Moreover, these nanoparticles generate reactive oxygen species (ROS) when exposed to water or moisture. ROS induces oxidative stress in bacterial cells, causing damage to proteins, lipids, and DNA. Accumulated ROS overwhelms cellular defense mechanisms, culminating in bacterial cell death. Acknowledging that the hydrogel matrix's absorption of resistance compounds might also play a role in the hydrogel's biodegradation or biocompatibility over time is vital. This absorption mechanism could not only diminish the accumulation of resistance compounds in the surroundings but also contribute to the prolonged effectiveness of the nanocomposite hydrogel as a potent antimicrobial agent. This comprehensive four-step pathway underscores the multifaceted nature of nanocomposite hydrogel antimicrobial activity, highlighting the intricate interplay between hydrogel matrices, metal oxide nanoparticles, bacterial cells, and their surrounding environment.

A remarkable facet of this paradigm is the potential for synergistic interactions between multiple growth factors. Nanocomposite hydrogels can house an ensemble of growth factors within the same or different nanoparticles, triggering a symphony of therapeutic effects that magnify their collective healing potential. Precision takes center stage as nanocomposite hydrogels can exert spatial and temporal control over the release of growth factors. This fine-tuned control paves the way for customized treatment regimens, where growth factors are dispatched at strategic wound healing or tissue regeneration junctures. The orchestration continues as bioactive nanoparticles within the nanocomposite hydrogel foster angiogenesis, the essence of new blood vessel formation. The consequential surge in nutrient and oxygen supply to healing tissues acts as a catalyst, hastening the healing process and bolstering tissue regeneration. The biomimetic nature of nanocomposite hydrogels further enriches the tapestry of benefits. These biomaterials mirror the intricate architecture of the natural extracellular matrix, beckoning cells to adhere, proliferate, and migrate with ease. Growth factors within nanoparticles only amplify these signals, transforming the hydrogel matrix into a nurturing milieu akin to the body's native tissue environment. Notorious for their delayed healing trajectory, chronic wounds stand to gain immensely from the sustained growth factor release orchestrated by nanocomposite hydrogels. These hydrogels effectively bridge the healing gap in challenging cases by providing an extended therapeutic intervention. Intriguingly, the customization of nanocomposite hydrogels for each patient's unique needs adds another layer of sophistication. Wound type, size, and individual healing dynamics are all factored in, rendering the treatment approach exquisitely tailored and optimized. Even the application process bears the hallmark of innovation, with nanocomposite hydrogels offering minimally invasive administration options. From injections to dressings, the patient's comfort is prioritized while ensuring seamless integration of this groundbreaking technology. The fusion of bioactive nanoparticles and nanocomposite hydrogels ushers in a new era for wound healing and tissue repair. As these advanced biomaterials synergistically leverage nanoparticles and hydrogel matrices, they orchestrate a harmonious delivery of growth factors, thus heralding a transformative chapter in wound care characterized by accelerated healing, facilitated tissue regeneration, and elevated standards of patient well-being.

Ahtzaz *et al.*^107^ used zinc oxide and zinc peroxide nanoparticles to improve angiogenesis and the formation of new blood vessels for tissue regeneration applications. Reactive oxygen species like hydrogen peroxide are known to promote angiogenesis. The researchers hypothesized that zinc peroxide, which has a higher oxidation potential than zinc oxide, would promote more angiogenesis. They created hydrogel scaffolds by crosslinking chitosan and cellulose polymers using triethyl orthoformate. They then embedded either zinc oxide or zinc peroxide nanoparticles into the hydrogel matrices. They characterized the nanoparticle-loaded hydrogels and tested their angiogenic potential using a chick chorioallantoic membrane assay. The results showed that the zinc peroxide-loaded hydrogel promoted significantly more angiogenesis than the zinc oxide hydrogel and the control hydrogel without nanoparticles. This suggests that the higher oxidation potential of zinc peroxide led to more on-site hydrogen peroxide production, which encouraged the formation of new blood vessels.

In summary, the study demonstrated that zinc peroxide nanoparticles, due to their higher oxidation potential, can promote more angiogenesis than zinc oxide nanoparticles. The zinc peroxide-loaded hydrogel scaffolds thus promise affordable biomaterials for tissue engineering applications requiring enhanced blood vessel formation. Engineered nanomaterials have great potential for improving wound healing and infection control. They can be used to develop wound dressings, drug delivery systems, and scaffolds for tissue regeneration. Researchers are exploring various types of nanomaterials like polymers, metals, ceramics, and clays. Chitosan nanoparticles have shown promise due to their inherent bioactivity, antimicrobial properties, and ability to deliver drugs and growth factors. Fibrin nanoparticles can also deliver growth factors and antibiotics to accelerate wound healing. Silver nanoparticles are effective against bacteria and fungi and can be incorporated into dressings and composites to control infections. Gold nanoparticles functionalized with antibiotics, antioxidants and growth factor genes have also shown potential for enhancing wound healing. Silica and clay nanoparticles can provide sustained release of therapeutics. They have been used to deliver nitric oxide, growth factors and genes to wounds. Zinc oxide nanoparticles mainly help with infection control. Nanomaterials combined with cells, drugs and biomolecules in composites and scaffolds have shown synergistic effects on wound healing. Researchers hope to develop multifunctional and multicomponent nanomaterials and 3D bioprinted structures to enhance wound healing. The focus is minimizing sepsis and modulating the immune response to infections. Overall, engineered nanomaterials promise to develop better treatments to accelerate wound healing and infection control.

## 8. Biocompatibility and immunomodulatory aspects of nanocomposite hydrogels

Nanocomposite hydrogels have emerged as innovative biomaterials with immense potential in various biomedical applications, from wound healing and tissue engineering to drug delivery and disease treatment. Central to their successful integration within the biological system is their biocompatibility-how well they interact with living tissues—and their immunomodulatory properties-how they influence immune responses. These critical considerations impact the safety, efficacy, and success of nanocomposite hydrogel-based therapies. Biocompatibility lies at the heart of any biomaterial's suitability for biomedical applications. Nanocomposite hydrogels must create a conducive environment for cells to thrive, proliferate, and contribute to the intended therapeutic outcome. For applications like wound healing and tissue engineering, it is paramount that the hydrogel matrix supports the viability and proliferation of relevant cells, such as fibroblasts or stem cells. The incorporation of nanoparticles should not compromise cellular health or functionality. The nanoparticles integrated into the hydrogel should exhibit minimal cytotoxicity to ensure they do not induce cell death or hinder cellular processes. Seamless integration with host tissues is crucial. Nanocomposite hydrogels should facilitate cellular infiltration and tissue regeneration without causing inflammation or rejection. Ideally, the hydrogel should trigger a controlled and appropriate immune response, promoting tissue repair rather than exacerbating inflammation. These considerations ensure that nanocomposite hydrogels function harmoniously within the biological milieu, encouraging therapeutic success while minimizing adverse effects. The immune system plays a pivotal role in the body's response to foreign materials, including nanocomposite hydrogels. Harnessing immunomodulation—the deliberate alteration of immune responses-can profoundly impact the efficacy of these biomaterials in various biomedical applications. Nanocomposite hydrogels can be tailored to possess anti-inflammatory properties. By incorporating anti-inflammatory nanoparticles or drugs, these hydrogels can help mitigate excessive immune responses that could hinder tissue repair. The interaction between nanoparticles and hydrogel matrices can influence macrophage polarization, guiding the immune response towards pro-regenerative phenotypes that support tissue healing. In specific scenarios where immunosuppression is beneficial, nanocomposite hydrogels can deliver immunomodulatory agents or nanoparticles that suppress immune reactions, such as in autoimmune disorders.

Conversely, nanocomposite hydrogels can stimulate the immune system for applications like cancer immunotherapy. This involves incorporating nanoparticles that enhance antigen presentation or immune cell activation. By engineering the hydrogel's composition and properties, researchers can impact the foreign body response—how the body reacts to implanted materials. This influences encapsulation around implants and long-term integration. Biocompatibility assessments must encompass potential allergic reactions to hydrogel components and nanoparticles to ensure patient safety. Understanding the long-term interaction of nanocomposite hydrogels with the immune system is essential, especially for chronic applications. A paradigm shift the combination of biocompatibility and immunomodulation in nanocomposite hydrogels represents a paradigm shift in biomedical applications. The ability to engineer hydrogels that interact harmoniously with the biological environment, promote healing, and influence immune responses offers a new dimension of precision in treatment strategies. Researchers can tailor hydrogel properties to modulate immune reactions, opening doors to personalized therapies that enhance tissue repair, combat diseases, and improve patient outcomes. As we continue to unravel the intricate interplay between nanocomposite hydrogels and the immune system, these biomaterials promise transformative advances across diverse fields of medicine.

Nanocomposite hydrogels possess the potential to induce regulatory T cell (Treg) responses, promoting immune tolerance and minimizing adverse reactions. By delivering immunomodulatory agents or presenting antigens within the hydrogel matrix, these materials encourage the generation of Tregs that dampen immune responses. This immune suppression can be harnessed for autoimmune disease treatment or allograft transplantation. The researchers in ref. 108 created nanofibrous gelatin/apatite composite scaffolds that mimic natural bone tissue's physical architecture and chemical composition. They developed a thermally induced phase separation technique to produce a nanofibrous gelatin matrix with a similar structure to natural collagen fibers. To make 3D scaffolds, they combined the phase separation method with a porogen leaching technique using paraffin spheres as templates. This created nanofibrous gelatin scaffolds with well-defined pores. Compared to commercial gelatin foam (Gelfoam), the nanofibrous scaffolds had a much higher surface area and mechanical strength. The researchers incorporated bone-like apatite particles onto the nanofibrous gelatin scaffolds to further improve the scaffolds using a simulated body fluid incubation process. This formed the composite scaffolds that mimic natural bone's nanoscale architecture and chemical composition.

**Fig. 10:**
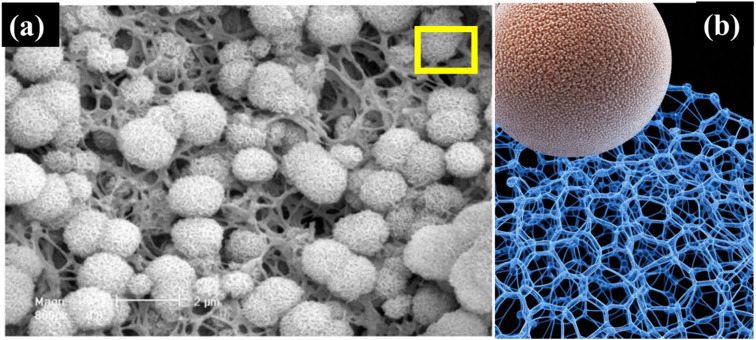
Figure displays scanning electron microscopy (SEM) images of NF-gelatin/apatite scaffolds subjected to incubation in a solution 1.5 times the strength of simulated body fluid (1.5 × SBF) for different durations. The image labeled “(a)” corresponds to the scaffold's appearance after 1 day of incubation. Image “(b)” illustrates that a significant apatite deposition is visible on the nanofibers of the NF-gelatin scaffold. The apatite deposits' surface coverage and size have tightened well, suggesting a progressive and successful incorporation of bone-like apatite onto the scaffold's surface. This image showcases the scaffold's surface and structure, highlighting any changes or interactions during incubation. The SEM micrograph provides valuable visual information about the progression of apatite incorporation onto the NF-gelatin scaffold over time, offering insights into the scaffold's bioactivity and potential for bone tissue engineering applications.

The nanofibrous gelatin/apatite composite scaffolds showed enhanced mechanical properties, osteoblast cell adhesion, proliferation, and differentiation compared to the nanofibrous gelatin scaffolds alone. So overall, the biomimetic composite scaffolds show promise for bone tissue engineering applications. In pursuit of enhancing both the mechanical robustness of the NF-gelatin scaffolds and promoting osteoblast differentiation, a novel strategy was employed. The aim was to integrate bone-like apatite onto the surface of NF-gelatin scaffolds through an *in situ* approach utilizing a simulated body fluid (SBF) technique. Upon close examination following a day of incubation, the surface of NF-gelatin pore walls displayed the presence of scattered and diminutive microparticles (as illustrated in Fig. 10). This intriguing observation signifies the initial stages of the apatite incorporation process onto the scaffold, holding the potential to fortify the scaffold's structural integrity while facilitating osteoblast differentiation for enhanced tissue regeneration.

## 9. Emerging trends and challenges in nanocomposite hydrogel research as promising biomaterials

Nanocomposite hydrogel research is a vibrant and rapidly evolving field situated at the captivating intersection of nanotechnology, materials science, and biomedicine. Within this dynamic realm, the continuous refinement of nanocomposite hydrogel capabilities is giving rise to novel trends and intricate challenges that shape the trajectory of scientific exploration and practical application. These emerging trends encompass a tapestry of innovation, including developing multi-functional nanocomposites that seamlessly integrate mechanical reinforcement, controlled drug delivery, and sensing capabilities, catalyzing groundbreaking advancements in smart therapeutics and diagnostics. The paradigm of personalized medicine further unfolds as nanocomposite hydrogels are meticulously tailored to match individual patient profiles, offering the prospect of precision drug administration, tissue engineering, and regenerative therapies, thereby capitalizing on the synergistic confluence of biomaterials, nanotechnology, and bioinformatics. Drawing inspiration from nature's ingenious designs, bioinspired nanocomposite hydrogels emulate the intricate architecture and composition of the extracellular matrix, nurturing enhanced cellular interactions, seamless tissue integration, and optimal overall performance. In a transformative convergence, integrating 3D printing and nanocomposite hydrogels empowers the precise fabrication of scaffold structures and nanoparticle distributions, a game-changing trend that shapes the landscape of tissue engineering, enabling the creation of intricate, functional constructs with unprecedented precision. The fusion of disparate nanoparticle types within nanocomposite hydrogels engenders hybrid systems with synergistic attributes, from metallic nanoparticles to drug-loaded polymeric counterparts, amplifying therapeutic potential across advanced therapies and diagnostics.

While these trends promise a profound impact, the journey is marked by formidable challenges. The pivotal concern of biocompatibility and safety underscores the need for rigorous assessment to ensure the long-term harmony between nanocomposite hydrogels and the intricate biological milieu, mainly when applied within the human body. Bridging the chasm between laboratory-scale production and large-scale manufacturing poses a significant hurdle, necessitating preserving quality and performance amidst scaling efforts. Navigating the intricate landscape of regulatory approvals necessitates a comprehensive grasp of established guidelines for medical devices and therapies involving nanocomposite hydrogels. As aspirations turn towards environmental consciousness, the quest for environmentally sustainable, biodegradable nanocomposite hydrogels is a pressing challenge, driving innovation toward ecologically sound solutions. The pivotal juncture of clinical translation mandates meticulous preclinical studies, rigorous testing, and impeccably designed clinical trials that validate the safety and efficacy of nanocomposite hydrogel applications. The complexity intrinsic to the interactions among nanoparticles, hydrogel matrices, and intricate biological systems underscores the imperative of interdisciplinary collaboration underpinned by advanced characterization techniques. A crucial concern entails ensuring nanocomposite hydrogels' enduring stability and unwavering performance across prolonged periods, particularly within the dynamic realms of physiological environments. Thus, as the journey of nanocomposite hydrogel research unfolds, these burgeoning trends and resolute challenges imprint an indelible mark, heralding an era of transformative innovation and paving the path toward ingenious solutions that redefine biomedical and materials applications.

## 10. Future perspectives and global implications of nanocomposite hydrogel innovations

The future of nanocomposite hydrogel innovations holds transformative potential that extends beyond current horizons, poised to redefine healthcare practices, forge global collaborations, and invoke profound ethical considerations. These innovations are set to emerge as pivotal catalysts in revolutionizing healthcare, ushering in an era characterized by personalized medical interventions, transcendent global cooperation, and meticulous ethical contemplation. Within this horizon of limitless possibilities, the following realms of future perspectives and global implications are illuminated:

(1) Nanocomposite hydrogels: architects of healthcare transformation a new dawn of personalized medicine: nanocomposite hydrogels are positioned as architects of a new dawn in personalized medicine, orchestrating treatments meticulously tailored to each patient's unique genetic makeup, health status, and therapeutic needs. Through their remarkable capacity to encapsulate an array of therapeutic agents and nanoparticles, these hydrogels have become the epicenter of precision medicine, crafting bespoke treatment regimens that optimize efficacy while minimizing potential side effects. Empowering remote and telemedicine frontiers: the advent of nanocomposite hydrogels wields a transformative influence on remote and telemedicine landscapes. By facilitating controlled and sustained drug release and real-time patient monitoring, these hydrogels empower healthcare providers to orchestrate interventions from afar, fine-tuning treatment modalities in response to evolving patient conditions. This infusion of advanced technology magnifies patient outcomes and alleviates burdens on healthcare systems, resonating with the essence of telemedicine's promise. Liberating patients through innovative therapeutics: nanocomposite hydrogels emerge as liberators of patients, endowing them with innovative therapeutic marvels that amplify the quality of life and foster individual autonomy. By granting access to sustained drug delivery, accelerated wound healing, and intricate tissue engineering strategies, these hydrogels pave avenues for self-care and holistic well-being, elevating patients from passive recipients to active participants in their healing journey.

(2) Nanotechnology and regenerative alchemy: a global collaboration unveiled global synergy in nanocomposite hydrogel research: the evolution of nanocomposite hydrogel innovations transcends geographical boundaries, cultivating a vibrant tapestry of global collaboration uniting scientists, engineers, and medical visionaries across continents. This symphony of collective wisdom expedites the exchange of knowledge, propagates best practices, and magnifies the collective impact of these innovations on the panorama of global healthcare. Bridgebuilders of healthcare disparities: nanocomposite hydrogels emerge as potent bridgebuilders, spanning chasms of healthcare disparities and erecting avenues of hope. These ingenious creations wield the potential to traverse the socioeconomic fabric, bestowing cost-effective, accessible, and resource-efficient solutions. By custom-tailoring interventions to suit specific medical challenges across diverse landscapes, these hydrogels democratize access to advanced biomedical technologies, painting a more egalitarian healthcare canvas. Championing healthcare equity through technological metamorphosis: the avant-garde tapestry of nanocomposite hydrogel innovations unfurls as champions of healthcare equity, pioneering the democratization of cutting-edge treatments. From facilitating wound healing amidst the remotest terrains to orchestrating targeted drug delivery in hitherto underserved communities, these innovations upend healthcare hierarchies, bestowing the gift of robust healthcare access and improved wellness to individuals worldwide.

(3) Ethical deliberations and societal discourse: a prerequisite for progress ethical quandaries in unleashing enhanced therapeutic prowess: as nanocomposite hydrogel technologies unleash augmented therapeutic prowess, they beckon forth a realm of ethical quandaries that warrant thoughtful introspection. The ethical vista embraces questions of informed patient consent, safeguarding individual privacy, and preempting unforeseen consequences, weaving an intricate tapestry of ethical contemplation that guides the judicious assimilation of these innovations into healthcare. Equitable dispensation of nanocomposite hydrogel benefits: a moral obligation unfurls to ensure that the myriad benefits borne of nanocomposite hydrogel innovations are disbursed equitably across diverse populations and geographic precincts. Collaborative endeavors, robust policy frameworks, and global partnerships converge to navigate this terrain, orchestrating a symphony of equitable access to these transformative solutions that transcend geographical, socioeconomic, and cultural boundaries. The pendulum of innovation and ethical prudence: as the horizon of nanocomposite hydrogel innovations expands, a delicate equilibrium beckons between audacious innovation and conscientious research and application. The critical interplay between pioneering advances and judicious safeguarding of human welfare mandates unwavering adherence to robust safety assessments, vigilant regulatory frameworks, and ongoing vigilance. This harmonious equilibrium endeavors to harness the boundless potential of nanocomposite hydrogel innovations while circumscribing potential risks. In crystallizing the panorama of future perspectives and global ramifications that unfurl from nanocomposite hydrogel innovations, a potent symphony of transformation takes center stage. As healthcare boundaries are redrawn, personalized medicine becomes an accessible reality, international collaborations surge, and ethical considerations guide progress. The tapestry of tomorrow's healthcare is intricately woven with nanocomposite hydrogel threads, interlacing the pursuit of well-being, the march of scientific inquiry, and the mantle of ethical stewardship into an indomitable symphony of holistic healthcare transformation that resonates across the globe.

## 11. Conclusion

Hydrogel biomaterials represent a groundbreaking frontier in biomedicine, providing innovative solutions to longstanding challenges in drug delivery, tissue engineering, and regenerative medicine. The strategic integration of nanotechnology with hydrogel chemistry facilitates the precise adjustment of physical, chemical, and biological properties, catering to intricate biomedical requirements. While natural biopolymers such as cellulose, chitosan, and alginate offer inherent biocompatibility and renewability, their limitations, like poor mechanical strength and rapid degradation, are mitigated by nanocomposite hydrogels. Strengthening the biopolymer matrix with nanoparticles enhances mechanical strength, improves biocompatibility, and enables modulation of drug release kinetics in response to stimuli. These nanocomposite hydrogels, mimicking the nanostructured architecture of the natural extracellular matrix, overcome traditional biomaterial limitations, minimizing foreign body responses and enhancing tissue integration. The combination of top-down nanofabrication and bottom-up self-assembly ensures unprecedented control over material structure and properties at the molecular level. Recent strides in the design, fabrication, and characterization of biopolymer-based nanocomposite hydrogels underscore their transformative potential, poised to address persistent challenges in biomedicine and usher in groundbreaking innovations in healthcare.

The realm of nanocomposite hydrogel innovations stands as a beacon of transformative potential, poised to redefine the landscape of healthcare and biomedical applications. These remarkable materials, born at the crossroads of nanotechnology, materials science, and biomedicine, catalyze a paradigm shift that transcends traditional boundaries and ushers in the future with promise and possibilities.

The journey through nanocomposite hydrogels has unveiled a multifaceted tapestry of applications, from advanced drug delivery and wound healing to regenerative medicine and diagnostic platforms. Their innate versatility, combined with the integration of nanoparticles, imparts them with unique and synergistic properties that unlock novel avenues for addressing complex healthcare challenges. With the potential to engineer personalized therapies, remotely manage patient conditions, and empower individuals to take charge of their health, nanocomposite hydrogels herald a new era where healthcare is tailored, accessible, and patient-centric.

This transformative potential is further magnified by the power of collaboration as international partnerships and interdisciplinary endeavors converge to accelerate knowledge exchange and amplify the collective impact of nanocomposite hydrogel research. These innovations permeate diverse socio-economic contexts and are crucial to bridging healthcare disparities and advancing biomedical solutions globally. However, this journey is not devoid of challenges. Ethical considerations loom, demanding thoughtful deliberations on patient consent, equitable access, and responsible innovation, striking a balance between groundbreaking research and ethical prudence imperative in steering the course of nanocomposite hydrogel applications toward a future that maximizes benefits while mitigating potential risks.

In the tapestry of healthcare transformation, nanocomposite hydrogel innovations are woven as threads of hope, resilience, and progress. They embody the resilience of scientific exploration, the spirit of collaboration, and the commitment to ethical integrity. As we stand at the cusp of this transformative journey, we are poised to witness the unfoldment of a healthcare landscape that is personalized, interconnected, and globally impactful. Nanocomposite hydrogels, with their boundless potential, are not just materials; they are the agents of change that can touch lives, improve well-being, and shape a healthier and more equitable world for future generations. Several promising future directions can be considered for advancing nanocomposite hydrogel research. First, developing multi-responsive nanocomposite hydrogels that react to multiple external stimuli, such as pH, temperature, and light, holds great potential for precise control over drug delivery and cell growth. Second, enhancing functionality by integrating more bioactive components like growth factors and stem cells could further tailor hydrogels for advanced tissue engineering and regenerative medicine. Third, combining 3D bioprinting techniques with nanocomposite hydrogels can facilitate the creation of patient-specific constructs for personalized medicine applications.

Focusing on nanofabrication tools and techniques also allows for controlling nanoscale features within hydrogels, mimicking the natural extracellular matrix. Techniques like offset printing of hydrogel patterns and advances in polymer and nanoparticle synthesis offer increased synthetic control, expanding the applicability of these materials. Moreover, advancements in molecular network engineering through supramolecular chemistry could lead to dynamically responsive hydrogels with reversible properties.

## Conflicts of interest

There are no conflicts to declare.

## Supplementary Material
